# Multicomponent Reactions Upon the Known Drug Trimethoprim as a Source of Novel Antimicrobial Agents

**DOI:** 10.3389/fchem.2019.00475

**Published:** 2019-07-04

**Authors:** Marina Pedrola, Marta Jorba, Eda Jardas, Ferran Jardi, Ouldouz Ghashghaei, Miguel Viñas, Rodolfo Lavilla

**Affiliations:** ^1^Laboratory of Medicinal Chemistry, Faculty of Pharmacy and Food Sciences and Institute of Biomedicine (IBUB), University of Barcelona, Barcelona, Spain; ^2^Laboratory of Molecular Microbiology & Antimicrobials, Department of Pathology & Experimental Therapeutics, Medical School, Hospitalet de Llobregat, University of Barcelona, Barcelona, Spain; ^3^Bellvitge Institute for Biomedical Research (IDIBELL), Barcelona, Spain

**Keywords:** antibiotics, drugs, isocyanides, multicomponent reactions, resistant bacteria

## Abstract

Novel antibiotic compounds have been prepared through a selective multicomponent reaction upon the known drug Trimethoprim. The Groebke-Blackburn-Bienaymé reaction involving this α-aminoazine, with a range of aldehydes and isocyanides afforded the desired adducts in one-step. The analogs display meaningful structural features of the initial drug together with relevant modifications at several points, keeping antibiotic potency and showing satisfactory antimicrobial profile (good activity levels and reduced growth rates), especially against methicillin-resistant *Staphylococcus aureus*. The new products may open new possibilities to fight bacterial infections.

## Introduction

**Figure 1:**
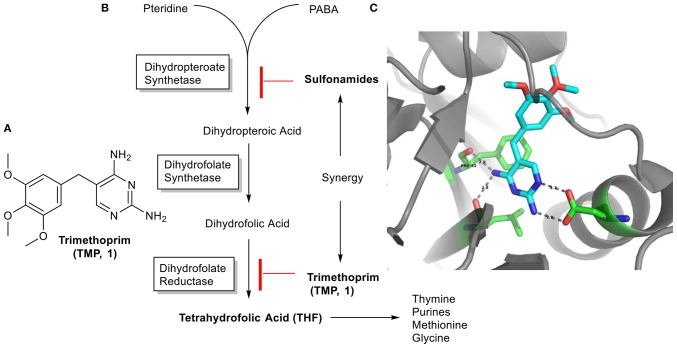
**(A)** TMP (**1**) structure. **(B)** Folic acid biosynthetic route. **(C)** Interactions of the TMP (**1**, in blue) with key amino acid residues at the DHFR binding site. Representation based on the 2W9H PDB file, with a resolution of 1.48 A (Heaslet et al., 2009).

Trimethoprim (TMP, **1**, Figure 1A) is a well-known antibiotic, present in the *Model List of Essential Medicines* from the World Health Organization. TMP is usually used in combinationwith Sulfamethoxazole (SMX) to treat lower urinary tract infections and acute invasive diarrhea/bacterial dysentery as first and second choice, respectively (WHO, 2017), respiratory infections in cystic fibrosis patients caused by *Staphylococcus aureus*, among other many infections. Lately, it has also been used for preventing infections from the opportunistic pathogen *Pneumocystis carinii* (Urbancic et al., 2018), which normally causes pneumonia in patients with AIDS. Both drugs act on the folic acid biosynthetic route by inhibiting two enzymes: dihydrofolate reductase (DHFR) and dihydropteroate synthetase, respectively. Folate needs to be synthesized by bacteria and it is crucial in the biosynthetic pathway of thymidine, essential in DNA synthesis. Hence, when used in combination, these antibiotics display a synergistic effect in inhibiting bacterial growth and leading to eventual cell death (Figure 1B) (Torok et al., 2009; Katzung et al., 2012).

The combination of sulfonamides and DHFR inhibitors has been clinically used since 1968 when it was first approved in the UK (Cody et al., 2008; Torok et al., 2009). Unfortunately, resistance emerged soon and has become widespread (Huovinen et al., 1995; Ventola, 2015). Nowadays, antibiotic resistance is one of the world's most pressing public health problems with high morbidity and mortality rates (Centers for Disease Control and Prevention, 2017). Furthermore, finding active drugs to fight both multidrug resistant infections and organisms is becoming extremely challenging, as is often the case of methicillin-resistant *Staphylococcus aureus* (MRSA) and multidrug resistant *Pseudomonas aeruginosa*. In this context, the co-therapy with TMP and SMX turns out to be ineffective to treat infections of a subset of bacteria with TMP-resistant DHFR enzymes (Heaslet et al., 2009).

Thus, the exploration of new synthetic compounds mimicking the structure and mechanism of action of conventional antimicrobial agents can be regarded as a main goal of this field of research. Such chemical entities would open new perspectives to reduce secondary effects and to enhance antimicrobial action and/or spectrum. Moreover, interaction between new molecules and conventional antimicrobials should be explored with the aim to find eventual synergistic activity.

**Scheme 1:**
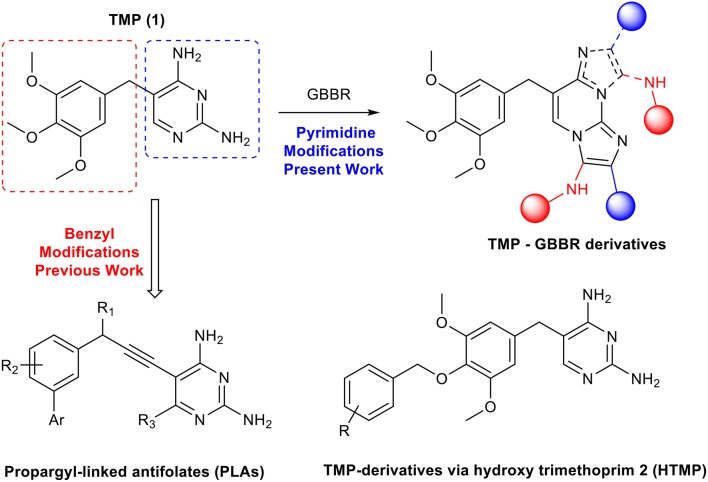
TMP-GBBR derivatives and precedent modifications on the benzyl moiety.

So far, some research groups have introduced chemical variability at the trimethoxybenzyl residue of the TMP in order to optimize the drug and improve its properties, overall activity and tackle TMP-resistance issues (Scheme 1) (Zhou et al., 2013; Lombardo et al., 2016; Rashid et al., 2016), being able to find promising potent compounds against *S. aureus* and *E. coli*. However, in this work we will focus our efforts on modifying the 2,4-diaminopyrimidine moiety, through a selective multicomponent reaction (MCR), the Groebke-Blackburn-Bienaymé reaction (GBBR). In the past, changes at this part of the molecule have not been considered, as they involve key interactions in the recognition of the natural substrate at the DHFR active site; however, the situation could be otherwise in mutated enzymes. Furthermore, inspection of the PDB of the crystal structure of a TMP-DHFR complex shows room for structural changes at the pyrimidine moiety (Figure 1C) (Heaslet et al., 2009). These GBBR-type modifications will change the heterocyclic core of the drug into a more complex di- or tricyclic azine, and up to four diversity points, which will be introduced in a regioselective manner (Scheme 1). These transformations, involving a relevant structural change on the TMP original framework, strictly speaking cannot be considered as drug late stage functionalizations (Cernak et al., 2016), although share the same philosophy of the approach.

MCRs represent an alternative to the usual sequential multistep synthesis. They involve the reaction of three or more starting materials in a convergent way to yield an adduct, whose structure embodies the major part of the reactive materials in a one-pot reaction through a unified mechanism (Zhu et al., 2014). Specifically, these reactions bring high efficiency, simplicity and both atom and step economy. Thus, they are of interest in medicinal chemistry to assemble relevant, complex heterocycles with remarkable bioactivities and to speed up the Structure-Activity Relationship studies (Hulme, 2005; Akritopoulou-Zanze and Djuric, 2010; Domling et al., 2012; Slobbe et al., 2012; Zarganes-Tzitzikas and Doemling, 2014).

**Scheme 2:**
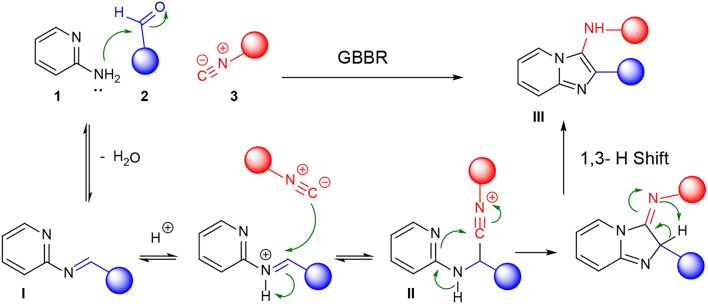
Mechanism of GBBR.

**Figure 2:**
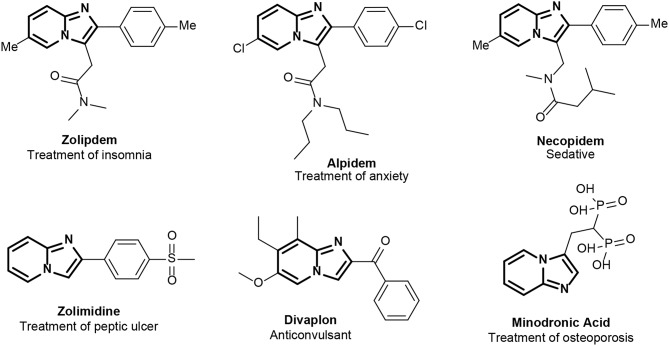
Example of commercial drugs with *N*-fused bicyclic imidazo-azines. The imidazo-azine core is highlighted in bold.

The GBBR (Scheme 2) is an isocyanide-based MCR yielding azine-fused imidazoles from readily available aldehydes (**2**), isocyanides (**3**) and amidine-type building blocks (**1**) (Bienaymé and Bouzid, 1998; Blackburn et al., 1998; Groebke et al., 1998). Several reviews have abstracted the main features of this MCR (Devi et al., 2015; Pericherla et al., 2015). This important transformation is massively used in medicinal chemistry, especially in drug discovery, because of the drug like character of the adduct (imidazo-azine), together with the power to decorate it with a wide range of structural diversity (Shaaban and Abdel-Wahab, 2016). These *N*-fused bicyclic imidazo-azines represent a special class of privileged scaffold found in several bioactive compounds and commercially available drugs, such as Zolpidem, Alpidem, Necopidem, Zolimidine, Divaplon, and Minodronic acid (approved for treatments of insomnia, anxiety, peptic ulcers, epilepsy, osteoporosis, etc.) (Figure 2). It is also well-known that α-polyamino-polyazines are important aromatic polyheterocycles present in a wide variety of clinical drugs, such as the antibacterial drug Trimethoprim, the anticonvulsant drug Lamotrigine and the anticancer drug Methotrexate. Furthermore, specific GBBR adducts have been identified as active antibiotics through phenotypic analyses, addressing a variety of targets (Al-Tel and Al-Qawasmeh, 2010; Shukla et al., 2012; Semreen et al., 2013; Kumar et al., 2014). These facts back our project to modify TMP via GBBR processes to deliver potentially useful novel antibiotics, either improving the activity of the original drug upon DHFR or acting through independent mechanisms.

## Results and Discussion

### Chemical Synthesis

**Scheme 3:**
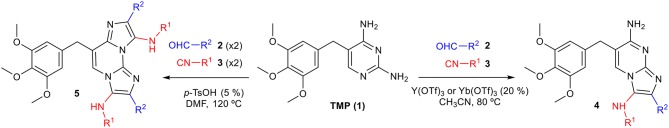
Synthesis of mono- (**4**) and double- (**5**) TMP GBBR adducts.

In this context, we planned to develop a series of TMP derivatives through the GBBR by interaction of the original drug (TMP, **1**) with a range of aldehydes (**2**) and isocyanides (**3**), and analyse the resulting MCR adducts as novel antibiotics, determining their potency, and efficiency, also considering their potential impact on resistant bacteria (Scheme 3).

The chemical modifications on TMP are based in our recent discoveries on GBBRs upon diaminopyrimidines, involving selective and multiple MCRs (Ghashghaei et al., 2018). In this way, the preparation of TMP analogs consisted in a regioselective mono-GBBR with an aldehyde/isocyanide pair, to yield derivatives **4**; it is worth mentioning that a kinetic control justifies the preferential formation of the observed isomer. Furthermore, double GBBR processes upon TMP yield doubly substituted derivatives **5**, with two equivalents of each reactant class (Scheme 3). The participation of a variety of Lewis acids catalyst is required to suitably generate and activate the imine intermediate and to achieve a moderate yield. In addition, standard flash chromatography purification was normally needed to afford the pure product. The designed analogs featured the *N*-fused bicyclic imidazo-azine scaffolds from the TMP reactant and displayed the variability points at substituents R^1^, derived from the isocyanide input (**3**) and R^2^ arising from the aldehyde reactant (**2**).

**Figure 3:**
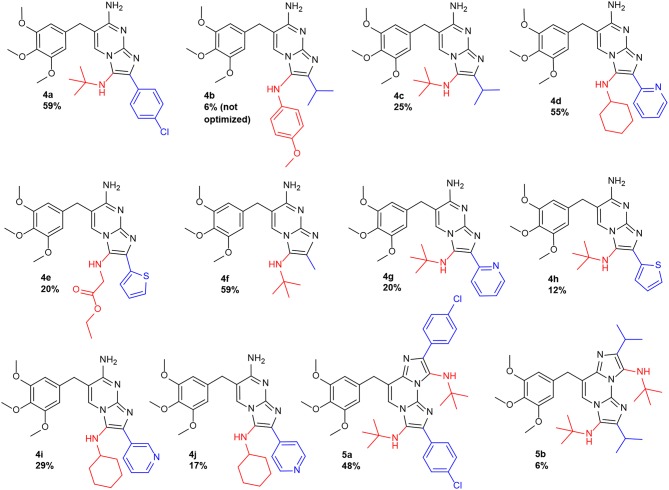
TMP mono**-** (**4**) and double**-**GBBR (**5**) adducts.

The processes worked in our TMP system as expected, yielding the corresponding products, showing the same reactivity and selectivity trends that were described in the unsubstituted diaminopyrimidine studies (Ghashghaei et al., 2018). For the initial screening, we prepared a series of TMP analogs featuring a variety of substituents on the imidazole amino group (R^1^, being *tert*-butyl, 4-methoxyphenyl, cyclohexyl, and ethoxycarbonylmethyl) whereas at its carbon position a range of aromatic or alkyl substituents were introduced (R^2^ being 4-chlorophenyl, α-, β-, or γ-pyridinyl, α-thienyl, methyl, and isopropyl). All the reactions were successful, yielding the mono-GBBR derivatives **4** and the doubly substituted-GBBR adducts **5** in acceptable yields (unoptimized). In this way, 12 new products (**4a**-**4j** and **5a-b**) arising from the corresponding aldehyde/isocyanide combinations were suitably prepared as pure materials (Figure 3).

The connectivity of the first analog synthesized (**4a**) was assigned through two-dimensional NMR experiments: HSQC, HMBC and NOESY spectra (see Supplementary Material) and matched with the expected structure, displaying the regioselectivity previously described (Ghashghaei et al., 2018). The rest of derivatives showed the same spectroscopical trends and their structures were assigned by analogy. Furthermore, the doubly substituted GBBR adducts **5** synthetically derived from the corresponding precursors **4**, then securing their identity.

**Scheme 4:**
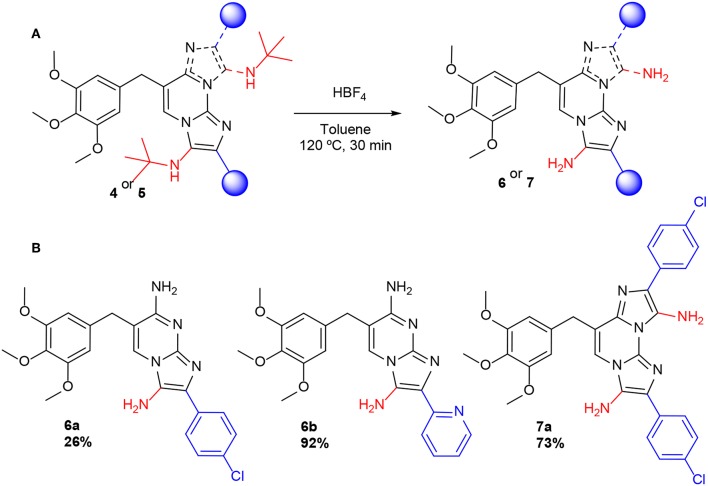
**(A)** Deprotection of N-*tert*-butyl groups in adducts **4** and **5**. **(B)** TMP-GBBR mono**-** (**6**) and double**-**adducts (**7**).

We planned to incorporate an unsubstituted amino group in the imidazole ring of the novel derivatives **4** and **5** in order to favor their recognition by the DHFR active site, in line with the natural substrate. Then, we tackled the preparation of such compounds through the acidic removal of a *tert*-butyl group from a suitable precursor adduct coming from MCRs involving *tert*-butyl isocyanide. Precedent work by Krasavin et al. (2008) demonstrated that this transformation is feasible in GBBR adducts. In this way, compounds **6a-6b** and **7a** were obtained as pure unsubstituted amino derivatives form *tert*-butyl precursors **4** and **5**, after HBF_4_ treatment (Scheme 4). All the synthesized compounds were suitably obtained in pure form, characterized and forwarded to microbiological analyses.

### Biological Analyses

The Minimum Inhibitory Concentration (MIC) values of the 15 TMP analogs against control strains are shown in Table 1 (for details, see the Supplementary Material). Although all the compounds showed MIC values against *E. coli* ATCC 25922 and *S. aureus* ATCC 29213 higher than TMP, some of them were almost as potent as TMP (**4c**, **4f**, **4h, 4i, 4j** and **6a**). *P. aeruginosa* PAO1 was found to be fully resistant to TMP, as well as to all new compounds. A preliminary inspection of the results showed that double GBBR adducts **5** lacked activity, probably meaning that they were unsuitable for binding to the target sites. Whereas for derivatives **4** some combinations were unproductive (especially the ones with aromatic and acetate R^1^ substituents and *p*-chlorophenyl group at R^2^ position), those featuring *tert*-butyl groups at R^1^ and isopropyl, methyl, β-, γ- (but not α-) pyridyl, and α-thienyl groups at R^2^ were particularly favored. Moreover, comparing compounds **4d**, **4g** and **6b**, we are able to confirm that the reduction of R^1^ substituents size allowed to decrease the MIC. It is also worthy to emphasize that all compounds resulted to be more active on *E. coli* than on *S. aureus*; **4i**, **4c**, and **4f** being the most potent ones. Thus, chemical modifications do not seem to limit the ability of the different new compounds to penetrate the outer membrane in Gram negative bacteria.

**Table 1:** Minimum Inhibitory Concentration (MIC, μM) of TMP and the new GBBR analogs against *E. coli* ATCC 25922, *S. aureus* ATCC 29213, and *P. aeruginosa* PAO1.

	**MIC (μM)**		
	*E. coli* ATCC 25922	*S. aureus* ATCC 29213	*P. aeruginosa* PAO1
TMP 1	0.43	13.78	> 110.22
4a	16.13	> 64.52	> 64.52
4b	> 67.01	> 67.01	> 67.01
4c	1.17	18.71	> 74.85
4d	8.19	> 65.50	> 65.50
4e	5.19	> 83.07	> 83.07
4f	1.25	80.10	> 80.10
4g	17.30	> 69.18	> 69.18
4h	2.14	> 68.44	> 68.44
4i	1.02	65.50	> 65.50
4j	2.05	65.50	> 65.50
5a	> 45.60	> 45.60	> 45.60
5b	> 56.66	> 56.66	> 56.66
6a	4.55	> 72.75	> 72.75
6b	2.46	78.73	> 78.73
7a	> 54.29	> 54.29	> 54.29

Almost all the new compounds acted synergistically with SMX as the control drug TMP did, against *E. coli* ATCC 25922 and *S. aureus* ATCC 29213 (Table 2); the latter species being much more sensitive to the SMX combination than to the treatment with the TMP-GBBR analogs alone. It also becomes apparent that nearly all the new compounds presented high activity against a set of clinical isolates of MRSA isolated from hospitalized or Cystic fibrosis (CF) patients. In CF patients, *Staphylococcus aureus* (and particularly MRSA) infection is the main challenge of antibiotic therapy, since the persistent infection caused by this bacterium is strongly associated with increased rates of decline in respiratory function and high mortality (Dolce et al., 2019). Thus, new approaches to fight this kind of bacterium are mandatory and should be based on new antimicrobials, most probably combined with conventional ones (Lo et al., 2018;Xhemali et al., 2019).

**Table 2:** Minimum Inhibitory Concentration (MIC, μM) of TMP and the new GBBR analogs in combination with Sulfamethoxazole (1:20) against *E. coli* ATCC 25922, *S. aureus* ATCC 29213, *P. aeruginosa* PAO1, *S. aureus* 8125304770, *S. aureus* 8139265926, *S. aureus* 8125255044, and *S. aureus* 8124825998.

	**MIC (μM)**						
	***E. coli* ATCC 25922**	***S. aureus* ATCC 29213**	***P. aeruginosa* PAO1**	***S. aureus* 8125304770**	***S. aureus* 8139265926**	***S. aureus* 8125255044**	***S. aureus* 8124825998**
TMP 1	0.11	0.43	13.78	0.431	0.86	0.22	0.43
4a	2.02	8.06	> 64.52	4.03	8.06	1.01	4.03
4b	67.01	> 67.01	> 67.01	67.01	> 67.01	67.01	> 67.00
4c	1.17	4.68	37.42	4.68	9.36	0.292	1.17
4d	2.05	4.09	> 65.50	4.09	4.093	1.02	4.09
4e	1.30	5.19	> 83.07	1.298	2.60	0.65	1.30
4f	0.63	5.01	40.05	0.63	2.50	0.63	1.25
4g	0.54	4.32	> 69.18	2.16	8.65	1.08	4.32
4h	0.27	2.14	34.22	1.07	1.07	0.27	1.07
4i	0.13	2.05	32.75	0.51	1.02	0.51	1.02
4j	0.26	2.05	32.75	1.02	2.05	0.51	1.02
5a	> 45.60	> 45.60	> 45.60	> 45.60	> 45.60	> 45.60	> 45.60
5b	28.33	> 56.66	> 56.66	28.331	> 56.66	14.17	56.66
6a	2.27	9.09	> 72.75	2.27	4.55	1.14	4.55
6b	0.31	2.46	78.73	1.23	2.46	0.62	2.46
7a	> 54.29	> 54.29	> 54.29	> 54.9	> 54.29	> 54.29	> 54.29

Again, derivatives **4c**, **4f**, **4h**, **4i**, **4j**, and **6b** were the most potent, but interestingly, some adducts which were not meaningful acting alone (Table 1), on SMX combination displayed a relevant potency (**4a**, **4d**, **4e**, **4g**, and **6a**). Disappointingly, no effect either of adducts alone or in combination with SMX was observed on *Pseudomonas aeruginosa* in any case, in line with the detected TMP activity. Particularly interesting are the activities against MRSA isolates as can be seen in Table 2, with many derivatives being as active as the TMP reference.

**Figure 4:**
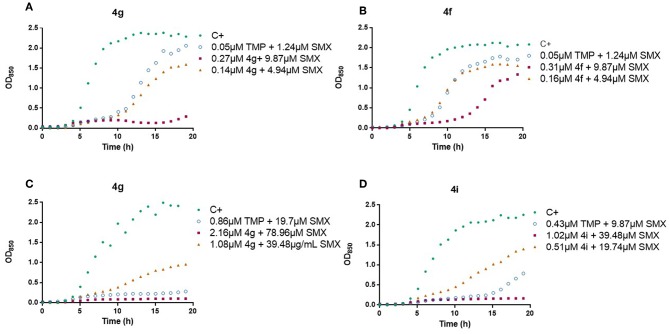
Effect of the analogs **4f**, **4g**, **4i** and TMP in combination with sulfamethoxazole (1:20) on the growth curve of *E. coli* (**4g**, **4f**; **A,B**) and *S. aureus* (**4g**, **4i**; **C,D**).

A relevant feature in the use of an antibiotic is its kinetic profile. Specifically, a fast reduction of the growth rates of the infective microorganism is of capital interest in therapeutics, arguably as important or more than the effective dose. Thus, the effect of TMP analogs in combination with SMX on the growth curves of *E. coli* ATCC 25922 and *S. aureus* ATCC 29213 was studied for the most interesting compounds (Figure 4 and Supplementary Material). Some differences were observed at subinhibitory concentrations of the antimicrobials (1/2 MIC and 1/4 MIC). In both tested bacteria, full inhibition occurred for derivatives **4g** and **4i** with SMX (1:20) at 1/2 the MIC value (Figures 4A,C,D). On the other hand, compounds **4g** and **4f**, at a concentration of 1/4 MIC, gave similar results than TMP at 1/2 MIC against *E. coli* ATCC 25922 (Figures 4A,B). In all the cases, significant reductions in the growth rates were observed when compared with the antimicrobial-free control, being comparatively better for some conditions than the TMP reference, especially compound **4i** for long culture times (Figure 4D).

## Conclusion

The marketed antibiotic TMP (**1**) has been succesfully modified by a GBB MCR with a range of commercially available aldehydes and isocyanides in selective processes yielding mono- or double- imidazo-azine adducts **4** and **5**. A short synthetic (one or two steps) protocol allowed access to a focused library of 15 TMP analogs featuring a novel heterocyclic scaffold with a relevant degree of chemical diversity at selected positions, including hydrogen atoms, small alkyl groups, aromatic and heteroaromatic rings. Incidentally, this work shows the possibility of using known drugs as substrates for MCRs and, in this manner, opens new ways to develop novel chemical entities of biological interest from this unusual origin. Antimicrobial activity of the novel analogs has been assayed in Grampositive (*S. aureus*) and Gramnegative (*E. coli*) microorganisms as well as on a bacterium considered the paradigm of resistance (*P. aeruginosa*). Despite the latter was resistant to all the new compounds, several mono-adducts **4** displayed MICs in the micromolar range against *E. coli* and *S. aureus*, what make us think that the TMP-derivatives bind to DHFR as well. The observed impact on growth kinetics allows us to conclude that the association of these new products with SMX exert a very similar effect than TMP itself. It is worthy to emphasize the excellent activities detected against MRSA strains. Given the reduced size of the focused chemset analyzed, and the relevant results found, we can conclude that the novel scaffold synthesized has potential to become a source for novel antibiotics. Further on going studies along these lines tackle toxicity, the mechanism of action and bacterial resistance issues.

## Data Availability

All datasets generated for this study are included in the manuscript and/or the Supplementary Files.

## Author Contributions

MP was responsible for designing and performing the initial experiments. EJ, FJ, OG, and RL performed the rest of the experimentation of the chemical section and analyzed the results. MJ designed and performed the microbiological experiments. MJ and MV analyzed the results of this section. All authors discussed the whole project and wrote the publication.

### Conflict of Interest Statement

The authors declare that the research was conducted in the absence of any commercial or financial relationships that could be construed as a potential conflict of interest. The reviewer FD declared a past co-authorship with one with the authors OG, RL to the handling editor.
