# Supplementary material for: Multicomponent Reactions Upon the Known Drug Trimethoprim as a Source of Novel Antimicrobial Agents
Source: Front Chem. 2019 Jul 4;7:475. doi: 10.3389/fchem.2019.00475 (PMC6621506; doi:10.3389/fchem.2019.00475)

Supplementary Material

Multicomponent Reactions upon the Known Drug Trimethoprim as a Source of Novel Antimicrobial Agents

Marina Pedrola [1], Marta Jorba [2], Eda Jardas [1], Ferran Jardi [1], Ouldouz Ghashghaei [1], Miguel Viñas[2] and Rodolfo Lavilla[1]

[1]Laboratory of Medicinal Chemistry, Faculty of Pharmacy and Food Sciences and Institute of Biomedicine (IBUB), University of Barcelona, Av. de Joan XXIII, 27-31, 08028 Barcelona, Spain

[2]Laboratory of Molecular Microbiology & Antimicrobials, Dept. Pathology & Experimental Therapeutics. Medical School. University of Barcelona and IDIBELL, Feixa Llarga, s/n. 08907 Hospitalet de Llobregat, Barcelona, Spain

Contents

Chemistry 3
General Information 3
Experimental Procedures for the Synthesis of Trimethoprim GBB Adducts and Related Compounds 4
Synthesis of Trimethoprim Mono GBBR Adducts 4
Synthesis of Trimethoprim Double GBBR Adducts 4
The tert-Butyl Removal from Trimethoprim GBBR Adducts 5
Characterization Data for the Isolated Compounds 6
Copies of the NMR Spectra 11
Microbiology 28
Material and Methods 28
Bacterial strains, bacteriological media and antimicrobials 28
Susceptibility testing 28
Determination of the Minimum Inhibitory Concentrations (MIC) 28
Effect of TMP-SMX and the GBBR analogues on bacterial growth curves 29
Growth Curves 29

Chemistry

General Information

Unless otherwise stated, all reactions were carried out under normal atmosphere in dried glassware. Commercially available reactants were used without further purification. Thin-layer chromatography was performed on pre-coated Merk silica gel 60 F_{254} plates and visualized under a UV lamp. Reactions were monitored by HPLC-MS using a HPLC Waters Alliance HT comprising a pump (Edwards RV12) with degasser, an autosampler and a diode array detector. Flow from the column was split to a MS spectrometer. The MS detector was configured with an electrospray ionization source (micromass ZQ4000) and nitrogen was used as the nebulizer gas. Data acquisition was performed with MassLynx software. When stated, the final crude was purified via flash column chromatography with a Combi Flash ISCO RF provided with dual UV detection. Prepacked normal phase silica or alumina gel columns (4, 12 and 24 g) were used for separation of products. ^{1}H and ^{13}C NMR spectra were recorded on a Varian Mercury 400 (at 400 MHz and 100 MHz, respectively). Unless otherwise quoted, NMR spectra were recorded in either $CDCl_3$ or DMSO solution with TMS as an internal reference. Data for ^{1}H NMR spectra are reported as follows: chemical shift (δ ppm), multiplicity, coupling constants (Hz) and integration. Data for ^{13}C NMR spectra are reported in terms of chemical shift (δ ppm). High Resolution Mass Spectrometry was performed by the University of Barcelona Mass Spectrometry Service. Yields correspond to the isolated pure compounds and the processes were not optimized.

Experimental Procedures for the Synthesis of Trimethoprim GBB Adducts and Related Compounds

Synthesis of Trimethoprim Mono GBBR Adducts

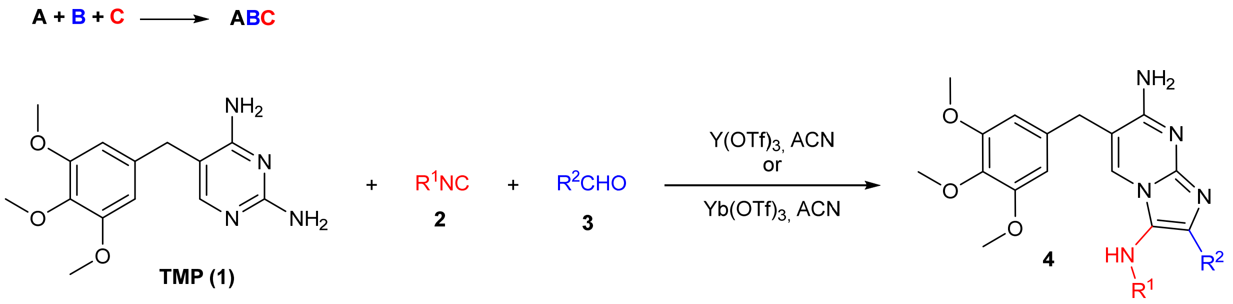

***General Procedure A (for synthesis of products 4a-4f)*:** A solution of trimethoprim (**1**, 1 mmol, 1 eq.) and aldehyde (**2**, 1 mmol, 1 eq.) in ACN (25 mL) was added into a Schlenk vessel followed by the addition of $Y(OTf)_3$ or $Yb(OTf)_3$ (0.2 mmol, 0.2 eq.) at room temperature. After 10 min, the suitable isocyanide (**3**, 1 mmol, 1 eq.) was added to the stirring reaction mixture; the vessel was closed and heated to 80 ºC overnight. After reaction completion was confirmed by TLC or HPLC, ACN was evaporated and DCM was added until everything was dissolved. The mixture was treated with saturated $NaHCO_3$ aqueous solution to basic pH. The organic layer was separated, dried over $MgSO_4$, filtered and evaporated under reduced pressure. The pure product **4** was obtained from the crude residue by flash chromatography with DCM/MeOH as eluent.

Synthesis of Trimethoprim Double GBBR Adducts

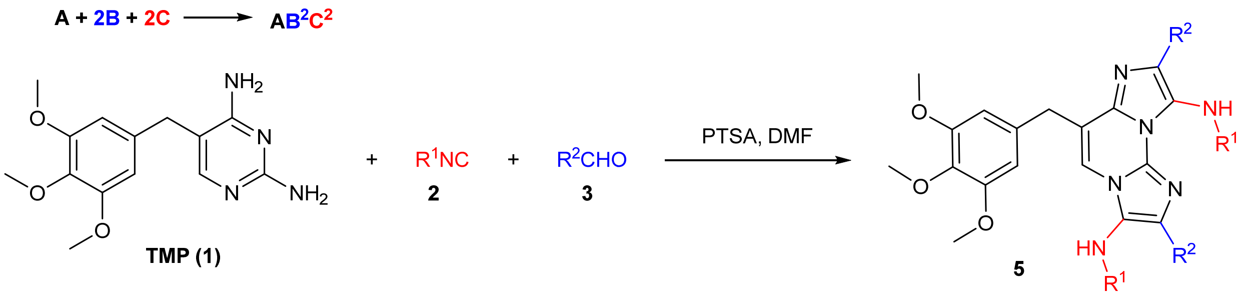

***General Procedure B (for synthesis of products 5a-5b)*:** A solution of trimethoprim (**1**, 1 mmol, 1 eq.) and aldehyde (**2**, 2 mmol, 2 eq.) in DMF (25 mL) was transferred to a Schlenk vessel followed by the addition of PTSA (0.2 mmol. 0.2 eq.) at room temperature. After 10 min, the suitable isocyanide (**3**, 2 mmol, 2 eq.) was added to the stirring reaction mixture, the vessel sealed and heated to 120 °C overnight. After reaction completion was confirmed by TLC or HPLC, solvent was evaporated and the mixture was treated with saturated $NaHCO_3$ aqueous solution to basic pH and extracted with DCM. The organic phase was dried over $MgSO_4$, filtered and evaporated under reduced pressure. The pure product **5** was obtained from the crude residue by flash chromatography with DCM/MeOH as eluent.

The tert-Butyl Removal from Trimethoprim GBBR Adducts

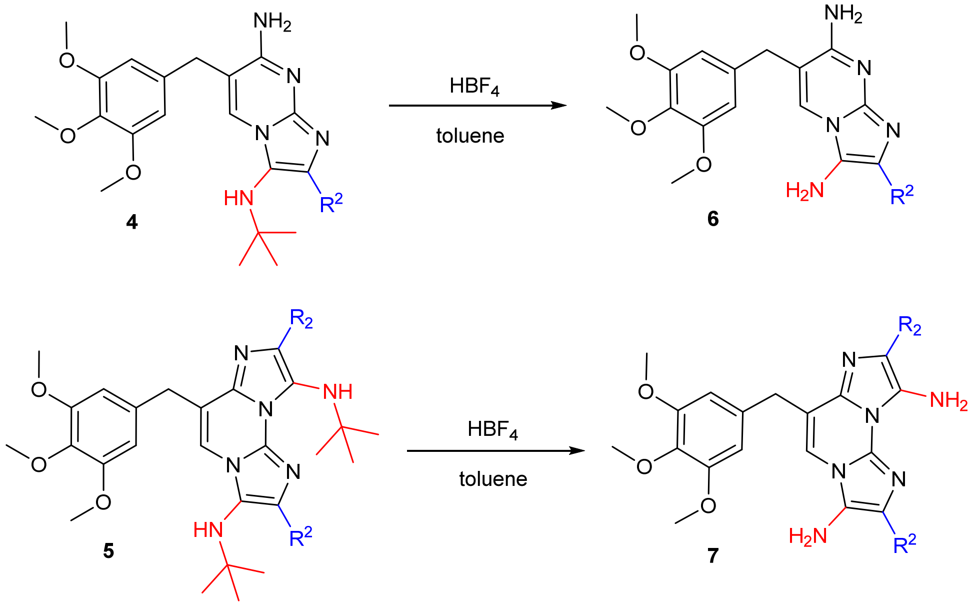

***General Procedure C (for synthesis of products 6)*:** To a solution of compound **4** (0.3 mmol, 1 eq.) in toluene (1.5 mL), tetrafluoroboric acid diethyl ether complex (0.15 mL, 1 mmol, 3 eq.) was added and reaction mixture was heated to 120 °C for 30 minutes. After reaction completion, confirmed by TLC or HPLC, the reaction mixture was cooled down to room temperature, treated with saturated $NaHCO_3$ aqueous solution to basic pH and extracted with ethyl acetate. The organic phase was dried over $MgSO_4$, filtered and evaporated under reduced pressure. The pure product **6** was obtained from the crude residue by flash chromatography with DCM/MeOH as eluent.

***General Procedure D (for synthesis of product 7)*:** To a solution of compound **5** (0.2 mmol, 1 eq.) in toluene (1.5 mL), tetrafluoroboric acid diethyl ether complex (0.5mL, 3.6 mmol, 18 eq.) was added and reaction mixture was heated to 120 °C for 30 minutes. After reaction completion, confirmed by TLC or HPLC, the reaction mixture was cooled down to room temperature, treated with saturated $NaHCO_3$ aqueous solution to basic pH and extracted with ethyl acetate. The organic phase was dried over $MgSO_4$, filtered and evaporated under reduced pressure. The pure product **7** was obtained by precipitation with dichloromethane.

Characterization Data for the Isolated Compounds

N^3-(tert-butyl)-2-(4-chlorophenyl)-6-(3,4,5-trimethoxybenzyl)imidazo[1,2-*a*]pyrimidine-3,7-diamine (4a)

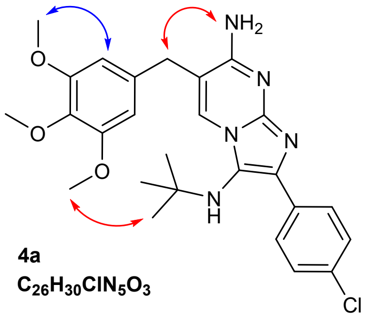

4a
$C_{26}H_{30}ClN_5O_3$

Following the *General Procedure A* with $Yb(OTf)_3$ as Lewis Acid, compound **4a** was obtained as a yellow solid, 59%. ^1H NMR (400 MHz, DMSO-d_6) δ 8.08 (d, J = 8.7 Hz, 2H), 7.81 (s, 1H), 7.38 (d, J = 8.7 Hz, 2H), 6.76 (s, 1H), 6.64 (s, 2H), 4.43 (s, 1H), 3.75 (s, 2H), 3.71 (s, 6H), 3.63 (s, 3H), 0.91 (s, 9H). ^{13}C NMR (101 MHz, DMSO-d_6) δ 157.76, 153.36, 145.21, 136.54, 134.44, 133.92, 131.18, 130.56, 129.26, 128.20, 121.30, 111.79, 106.83, 60.42, 56.21, 55.58, 34.04, 30.36. One C signal was not detected. HRMS: calcd for $C_{26}H_{31}ClN_5O_3$ 496.2110 (M+H$^+$); found 496.2125. For this molecule, NOESY, HMBC and HSQC spectra were analyzed: positive control crosspeak is marked in blue, diagnostic interactions are marked in red.

2-isopropyl-N^3-(4-methoxyphenyl)-6-(3,4,5-trimethoxybenzyl)imidazo[1,2-*a*]pyrimidine-3,7-diamine (4b)

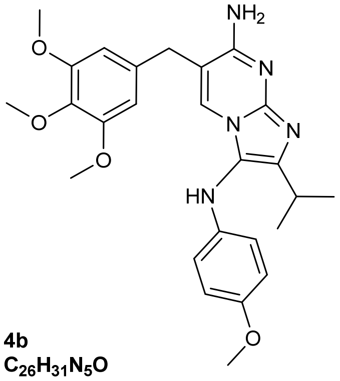

4b
$C_{26}H_{31}N_5O$

Following the *General Procedure A* with $Yb(OTf)_3$ as Lewis Acid, compound **4b** was obtained as a light yellow solid, 6% (unoptimized). ^1H NMR (400 MHz, $CDCl_3$) δ 7.53 (s, 1H), 6.71 (d, J = 8.8 Hz, 2H), 6.46 (d, J = 8.9 Hz, 2H), 6.30 (s, 2H), 5.33 (s, 1H), 3.80 (s, 3H), 3.73 (s, 6H), 3.72 (s, 3H), 3.69 (s, 2H), 3.13 – 2.93 (m, 1H), 2.43 (s, 2H), 1.28 (d, J = 6.9 Hz, 6H). ^{13}C NMR (101 MHz, $CDCl_3$) δ 157.06, 153.80, 153.38, 146.25, 145.92, 139.88, 137.23, 132.30, 129.63, 115.11, 114.59, 114.10, 109.73, 105.37, 60.99, 56.25, 55.78, 35.34, 26.69, 22.37. HRMS: calcd for $C_{26}H_{32}N_5O$ 478.2449 (M+H$^+$); found 478.2455.

N^3-(tert-butyl)-2-isopropyl-6-(3,4,5-trimethoxybenzyl)imidazo[1,2-*a*]pyrimidine-3,7-diamine (4c)

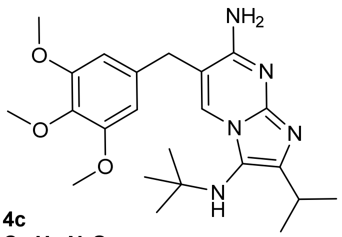

4c
$C_{23}H_{33}N_5O_3$

Following the *General Procedure A* with $Yb(OTf)_3$ as Lewis Acid, compound **4c** was obtained as a light brown solid, 25%. ^1H NMR (400 MHz, $CDCl_3$) δ 7.79 (s, 1H), 6.40 (s, 2H), 4.95 (s, 1H), 3.83 (s, 3H), 3.79 (s, 6H), 3.78 (s, 3H), 3.07 – 2.94 (m, 1H), 2.64 (s, 1H), 1.31 (d, J = 6.8 Hz, 6H), 1.13 (s, 9H). ^{13}C NMR (101 MHz, $CDCl_3$) δ 156.68, 154.19, 145.73, 137.56, 133.04, 130.98, 118.72, 109.42, 105.78, 61.34, 56.59, 55.10, 35.82, 30.62, 26.41, 23.11. One C signal was not detected. HRMS: calcd for $C_{23}H_{34}N_5O_3$ 428.2656 (M+H$^+$);

found 428.2674.

N^3-cyclohexyl-2-(pyridin-2-yl)-6-(3,4,5-trimethoxybezyl)imidazo[1,2-*a*]pyrimidine-3,7-diamine (4d)

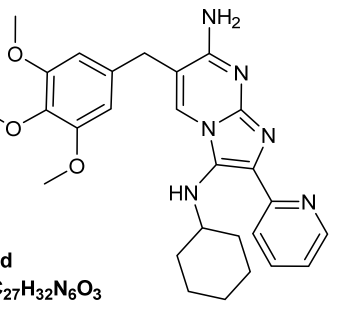

4d
$C_{27}H_{32}N_6O_3$

Following the *General Procedure A* with $Y(OTf)_3$ as Lewis Acid, compound **4d** was obtained as brown solid, 55%. ^1H NMR (400 MHz, $CDCl_3$) δ 8.46 (d, *J* = 4.2 Hz, 1H), 8.07 (d, *J* = 8.0 Hz, 1H), 7.67 – 7.61 (m, 2H), 7.04 – 7.00 (m, 1H), 6.41 (s, 2H), 5.98 (s, 1H), 5.45 (s, 2H), 5.27 (s, 1H), 3.82 (s, 3H), 3.79 (s, 8H), 2.83 (s, 1H), 1.79 (d, *J* = 12.3 Hz, 2H), 1.68 (s, 2H), 1.52 (s, 1H), 1.22 (dd, *J* = 23.1, 12.1 Hz, 3H), 1.14 (t, *J* = 9.2 Hz, 2H). ^{13}C NMR (101 MHz, $CDCl_3$) δ 156.49, 155.20, 153.82, 148.24, 144.02, 137.19, 136.41, 132.23, 129.95, 128.57, 127.57, 120.54, 120.21, 110.83, 105.60, 60.93, 56.75, 56.20, 35.35, 33.98, 25.77, 25.00. HRMS: calcd for $C_{27}H_{33}N_6O_3$ 489.2609 (M+H+); found 489.2604.

Ethyl(7-amino-2-(thiophen-2-yl)-6-(3,4,5-trimethoxybezyl)imidazo[1,2-*a*]pyrimidin-3-yl)glycinate (4e)

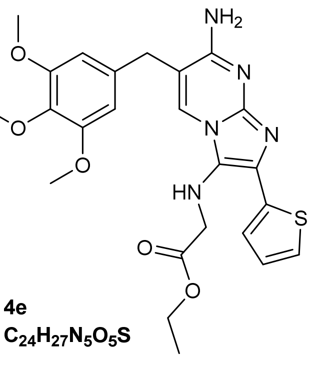

4e
$C_{24}H_{27}N_5O_5S$

Following the *General Procedure A* with $Y(OTf)_3$ as Lewis Acid, compound **4e** was obtained as a brown solid, 20%. ^1H NMR (400 MHz, $CDCl_3$) δ 8.12 (s, 1H), 7.53 (dd, *J* = 3.6, 1.1 Hz, 1H), 7.24 (dd, *J* = 5.1, 1.1 Hz, 1H), 7.05 (dd, *J* = 5.1, 3.6 Hz, 1H), 6.42 (s, 2H), 5.45 (s, 2H), 4.15 – 4.09 (m, 2H), 3.81 (s, 3H), 3.78 (s, 6H), 3.77 (s, 2H), 2.15 (s, 2H), 1.21 (dd, *J* = 8.0, 6.2 Hz, 3H). ^{13}C NMR (101 MHz, $CDCl_3$) δ 171.87, 157.22, 153.28, 144.89, 136.71, 136.42, 132.28, 130.41, 129.94, 127.34, 124.25, 123.99, 120.66, 109.86, 106.23, 60.82, 60.58, 55.88, 49.16, 34.97, 29.04. HRMS: calcd for $C_{24}H_{28}N_5O_5S$ 498.1806 $(M+H^+)$; found 498.1806.

N^3-(*tert*-butyl)-2-methyl-6-(3,4,5-trimethoxybenzyl)imidazo[1,2-*a*]pyrimidine-3-7-diamine (4f)

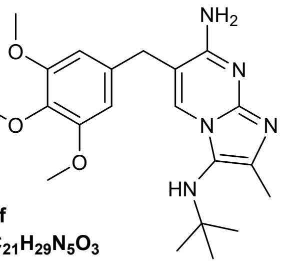

4f
$C_{21}H_{29}N_5O_3$

Following the *General Procedure A* with $Yb(OTf)_3$ as Lewis Acid, compound **4f** was obtained as a white powder, 59%. ^1H NMR (400 MHz, DMSO- d_6) δ 7.11 (s, 1H), 6.62 (s, 2H), 6.46 (s, 1H), 3.71 (s, 8H), 3.64 (s, 3H), 3.38 (s, 2H), 2.12 (s, 3H), 1.00 (s, 9H). ^{13}C NMR (101 MHz, DMSO-d_6) δ 156.70, 153.30, 144.83, 136.45, 134.34, 133.12, 130.13, 120.67, 110.32, 106.74, 60.40, 56.18, 54.87, 34.00, 30.18, 14.07. HRMS: calcd for $C_{21}H_{30}N_5O_3$ 400.2270 $(M+H^+)$; found 400.2338.

N^3-(*tert*-butyl)-2-(pyridin-2-yl)-6-(3,4,5-trimethoxybenzyl)imidazo[1,2-*a*]pyrimidine-3,7-diamine (4g)

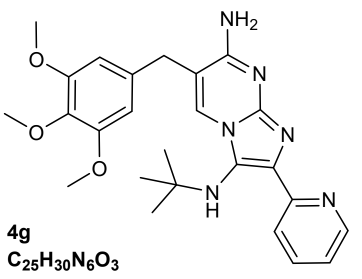

4g
$C_{25}H_{30}N_6O_3$

Following the *General Procedure A* with $Yb(OTf)_3$ as Lewis Acid, compound **4g** was obtained as a green solid, 20%. ^{1}H NMR (400 MHz, DMSO-d$_6$) δ 8.50 (d, *J* = 4.0 Hz, 1H), 8.16 (d, *J* = 8.0 Hz, 1H), 7.90 (s, 1H), 7.72 (t, *J* = 7.6 Hz, 1H), 7.15 – 7.04 (m, 1H), 6.43 (s, 2H), 5.53 (s, 2H), 3.84 (s, 3H), 3.81 (s, 8H), 2.31 (s, 1H), 1.09 (s, 9H). ^{13}C NMR (101 MHz, DMSO-d$_6$) δ 164.60, 157.72, 153.07, 148.52, 144.24, 136.74, 136.24, 133.28, 130.15, 125.20, 121.55, 120.79, 112.66, 106.59, 60.09, 55.94, 55.89, 33.60, 29.69. HRMS: calcd for $C_{25}H_{31}N_6O_3$ 463.2379 (M+H$^+$); found 463.2445.

N^3-(*tert*-butyl)-2-(thiphen-2-yl)-6-(3,4,5-trimethoxybenzyl)imidazo[1,2-*a*]pyrimidine-3,7-diamine (4h)

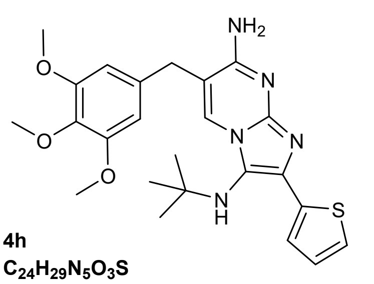

4h
$C_{24}H_{29}N_5O_3S$

Following the *General Procedure A* with $Yb(OTf)_3$ as Lewis Acid, compound **4h** was obtained as a yellow solid, 12%. ^{1}H NMR (400 MHz, $CDCl_3$) δ 7.69 (s, 1H), 7.57 (s, 1H), 7.18 (s, 1H), 6.99 (dd, *J* = 4.8, 3.8 Hz, 1H), 6.38 (s, 2H), 5.32 (s, 1H), 3.78 (s, 3H), 3.75 (s, 9H), 2.90 (s, 1H), 1.05 (s, 9H). ^{13}C NMR (101 MHz, DMSO-d$_6$) δ 156.93, 152.93, 144.88, 138.71, 136.08, 133.48, 131.16, 129.98, 127.10, 124.05, 123.50, 119.32, 110.98, 106.42, 59.97, 55.76, 55.23, 33.64, 30.12. HRMS: calcd for $C_{24}H_{30}N_5O_3S$ 468.1991 (M+H$^+$); found 468.2054.

N^3-cyclohexyl-2-(pyridin-3-yl)-6-(3,4,5-trimethoxybenzyl)imidazo[1,2-*a*]pyrimidine-3,7-diamine (4i)

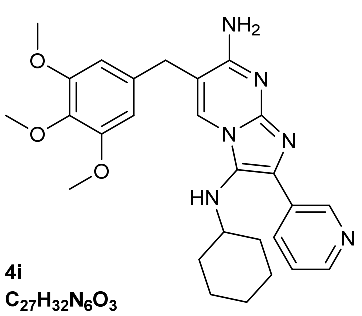

4i
$C_{27}H_{32}N_6O_3$

Following the *General Procedure A* with $Yb(OTf)_3$ as Lewis Acid, compound **4i** was obtained as a pale yellow solid, 29%. ^{1}H NMR (400 MHz, DMSO-d$_6$) δ 9.26 (d, *J* = 1.8 Hz, 1H), 8.38 (dd, *J* = 4.8, 1.5 Hz, 1H), 8.37 – 8.34 (m, 1H), 7.73 (s, 1H), 7.38 (dd, *J* = 7.9, 4.8 Hz, 1H), 6.72 (s, 2H), 6.68 (s, 2H), 4.61 (d, *J* = 5.2 Hz, 1H), 3.76 (d, *J* = 4.0 Hz, 2H), 3.75 (s, 6H), 3.66 (s, 3H), 2.68 (d, *J* = 4.3 Hz, 1H), 1.71 – 1.43 (m, 5H), 1.20 – 0.99 (m, 5H). ^{13}C NMR (101 MHz, DMSO-d$_6$) δ 157.04, 152.89, 147.14, 146.74, 144.84, 136.04, 133.43, 132.65, 130.83, 129.38, 129.23, 123.25, 123.00, 111.59, 106.35, 59.88, 56.63, 55.72, 33.59, 33.43, 25.35, 24.34. HRMS: calcd for $C_{27}H_{33}N_6O_3$ 489.2536 (M+H$^+$); found 489.2603.

***N*[3]-cyclohexyl-2-(pyridine-4-yl)-6-(3,4,5-trimethoxybenzyl)imidazo[1,2-*a*]pyrimidine-3,7-diamine (4j)**

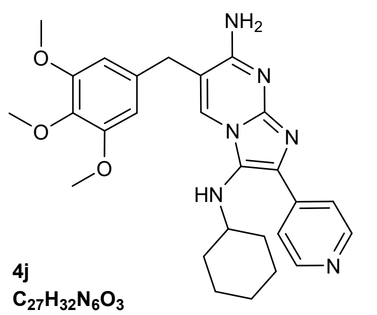

4j
$C_{27}H_{32}N_6O_3$

Following the *General Procedure A* with $Yb(OTf)_3$ as Lewis Acid, compound **4j** was obtained as a yellow solid, 17%. ^{1}H NMR (400 MHz, DMSO-d_6) δ 8.50 (d, *J* = 6.1 Hz, 2H), 7.97 (dd, *J* = 4.7, 1.6 Hz, 2H), 7.73 (s, 1H), 6.75 (s, 2H), 6.67 (s, 2H), 4.66 (d, *J* = 5.8 Hz, 1H), 3.75 (s, 2H), 3.74 (s, 6H), 3.65 (s, 3H), 2.76 – 2.58 (m, 1H), 1.60 (dd, *J* = 43.6, 20.4 Hz, 5H), 1.24 – 1.02 (m, 5H). ^{13}C NMR (101 MHz, DMSO-d_6) δ 157.73, 153.39, 149.91, 145.22, 142.58, 136.54, 133.82, 129.72, 129.41, 125.64, 120.50, 112.52, 106.83, 60.39, 57.33, 56.21, 34.08, 33.97, 25.81, 24.92. HRMS: calcd for $C_{27}H_{33}N_6O_3$ 489.2536 (M+H$^+$); found 489.2600.

***N*[3],*N*[9]-di-tert-butyl-2,8-bis(4-chlorophenyl)-6-(3,4,5-trimethoxybenzyl)diimidazo[1,2-*a*:1',2'-*c*]pyrimidine-3,9-diamine (5a)**

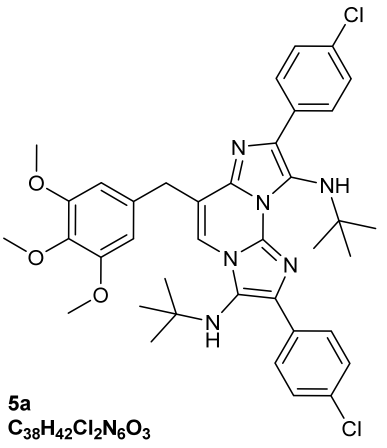

5a
$C_{38}H_{42}Cl_2N_6O_3$

Following the *General Procedure B*, compound **5a** was obtained as an orange solid, 48%. ^{1}H NMR (400 MHz, $CDCl_3$) δ 8.18 (d, *J* = 8.5 Hz, 2H), 7.90 (d, *J* = 8.5 Hz, 2H), 7.37 (dd, *J* = 8.5, 6.4 Hz, 4H), 7.14 (s, 1H), 6.64 (s, 2H), 4.17 (s, 2H), 3.86 (s, 3H), 3.84 (s, 6H), 1.26 (s, 1H), 1.14 (s, 9H), 1.00 (s, 9H). ^{13}C NMR (101 MHz, $CDCl_3$) δ 153.51, 137.75, 136.99, 134.92, 134.31, 133.82, 133.68, 133.06, 132.89, 132.70, 132.34, 129.21, 128.61, 128.46, 128.35, 123.04, 118.49, 116.75, 106.77, 61.03, 58.10, 56.22, 56.05, 34.25, 30.55, 30.18. One C signal not detected. HRMS: calcd for $C_{38}H_{43}Cl_2N_6O_3$ 701.2768 (M+H$^+$); found 701.2785.

***N*[3],*N*[9]-di-tert-butyl-2,8-diisopropyl-6-(3,4,5-trimethoxybenzyl)diimidazo[1,2-*a*:1',2'-*c*]pyrimidine-3,9-diamine (5b)**

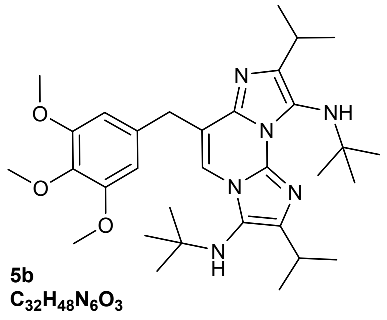

5b
$C_{32}H_{48}N_6O_3$

Following the *General Procedure B*, compound **5b** was obtained as a light brown solid, 6%. ^{1}H NMR (400 MHz, $CDCl_3$) δ 7.11 (s, 1H), 6.65 (s, 2H), 4.10 (s, 2H), 3.83 (s, 3H), 3.82 (s, 6H), 3.30-3.23 (m, 1H), 3.02-2.93 (m, 1H), 2.53 (s, 1H), 1.73 (s, 1H), 1.35 (d, *J* = 6.9 Hz, 6H), 1.25 (d, *J* = 6.5 Hz, 6H), 1.24 (s, 9H) 1.08 (s, 9H). ^{13}C NMR (101 MHz, $CDCl_3$) δ 153.11, 143.03, 139.72, 137.45, 136.52, 134.67, 134.58, 126.18, 120.26, 117.71, 115.44, 106.43, 60.79, 55.97, 55.48, 54.19, 34.25, 30.17, 29.55, 25.81, 25.60, 22.98, 22.81. HRMS: calcd for $C_{32}H_{49}N_6O_3$ 565.3861

(M+H+); found 565.3858.

2-(4-chlorophenyl)-6-(3,4,5-trimethoxybenzyl)imidazo[1,2-*a*]pyrimidine-3,7-diamine (6a)

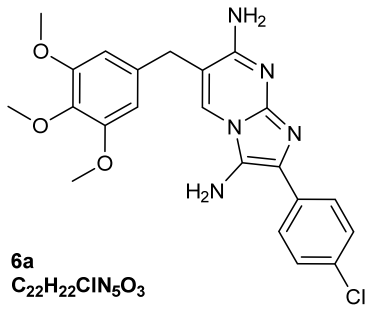

6a
$C_{22}H_{22}ClN_5O_3$

Following the *General Procedure C*, compound **6a** was obtained as a yellow solid, 26%. ^{1}H NMR (400 MHz, DMSO-d$_6$) δ 8.01 (s, 1H), 7.99 (d, *J* = 8.7 Hz, 2H), 7.39 (d, *J* = 8.7 Hz, 2H), 6.65 (s, 2H), 4.72 (s, 2H), 3.77 (s, 2H), 3.74 (s, 6H), 3.64 (s, 3H). HRMS: calcd for $C_{22}H_{23}ClN_5O_3$ 440.1484 (M+H$^+$); found 440.1483.

2-(pyridin-2-yl)-6-(3,4,5-trimethoxybenzyl)imidazo[1,2-*a*]pyrimidine-3,7-diamine (6b)

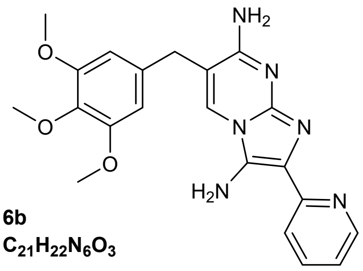

6b
$C_{21}H_{22}N_6O_3$

Following the *General Procedure C*, compound **6b** was obtained as a yellow solid, 92%. There is a 15% of impurity. ^{1}H NMR (400 MHz, DMSO-d$_6$) δ 8.46 (ddd, *J* = 4.9, 1.7, 0.9 Hz, 1H), 7.90 (s, 1H), 7.82 – 7.67 (m, 2H), 7.01 (ddd, *J* = 7.2, 4.9, 1.3 Hz, 1H), 6.65 (s, 2H), 6.51 (s, 2H), 6.00 (d, *J* = 22.6 Hz, 2H), 3.74 (s, 6H), 3.73 (s, 2H), 3.64 (s, 2H). ^{13}C NMR (101 MHz, DMSO-d$_6$) δ 156.15, 155.86, 152.88, 148.29, 142.00, 136.32, 136.14, 133.74, 129.13, 128.79, 118.62, 118.17, 110.82, 106.30, 106.07, 59.96, 55.89, 33.81. HRMS: calcd for $C_{21}H_{23}N_6O_3$ 407.1753 (M+H$^+$); found 407.1810 as a minor peak. The major peak corresponded to $C_{22}H_{26}N_6O_4$ (M+MeOH$^+$), calcd 437.1937); found 437.1926.

2,8-bis(4-chlorophenyl)-6-(3,4,5-trimethoxybenzyl)diimidazo[1,2-*a*:1',2'-*c*]pyrimidine-3,9-diamine (7a)

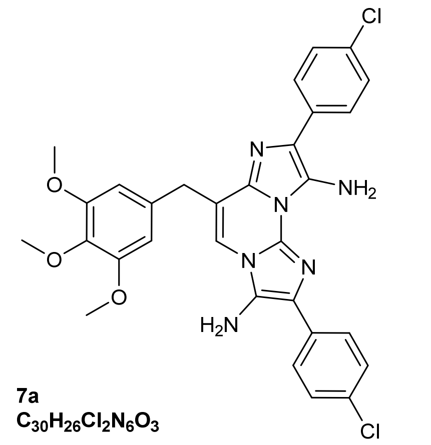

7a
$C_{30}H_{26}Cl_2N_6O_3$

Following the *General Procedure D*, compound **7a** was obtained as light yellow solid, 73%. ^{1}H NMR (400 MHz, DMSO-d$_6$) δ 7.97 (dd, *J* = 9.7, 8.9 Hz, 4H), 7.76 (s, 1H), 7.46 (dd, *J* = 9.7, 8.8 Hz, 4H), 6.86 (s, 2H), 6.38 (s, 2H), 5.28 (s, 2H), 4.02 (s, 2H), 3.76 (s, 6H), 3.62 (s, 3H). ^{13}C NMR (101 MHz, DMSO-d$_6$) δ 152.63, 136.04, 134.67, 133.56, 133.40, 133.24, 132.39, 131.74, 129.38, 129.23, 128.35, 128.27, 126.91, 126.57, 126.15, 119.90, 118.91, 116.66, 115.04, 106.54, 59.92, 55.79, 54.90. HRMS: calcd for $C_{30}H_{27}Cl_2N_6O_3$ 589.1516 (M+H$^+$); found 589.1539.

Copies of the NMR Spectra

N^3-(tert-butyl)-2-(4-chlorophenyl)-6-(3,4,5-trimethoxybenzyl)imidazo[1,2-*a*]pyrimidine-3,7-diamine (4a)

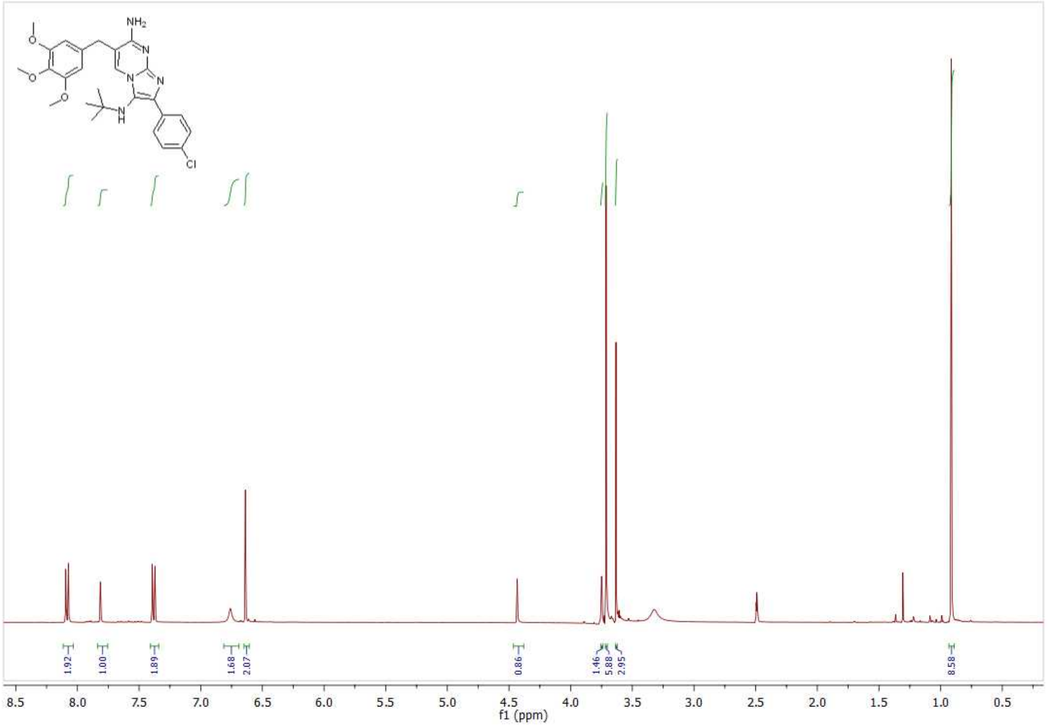

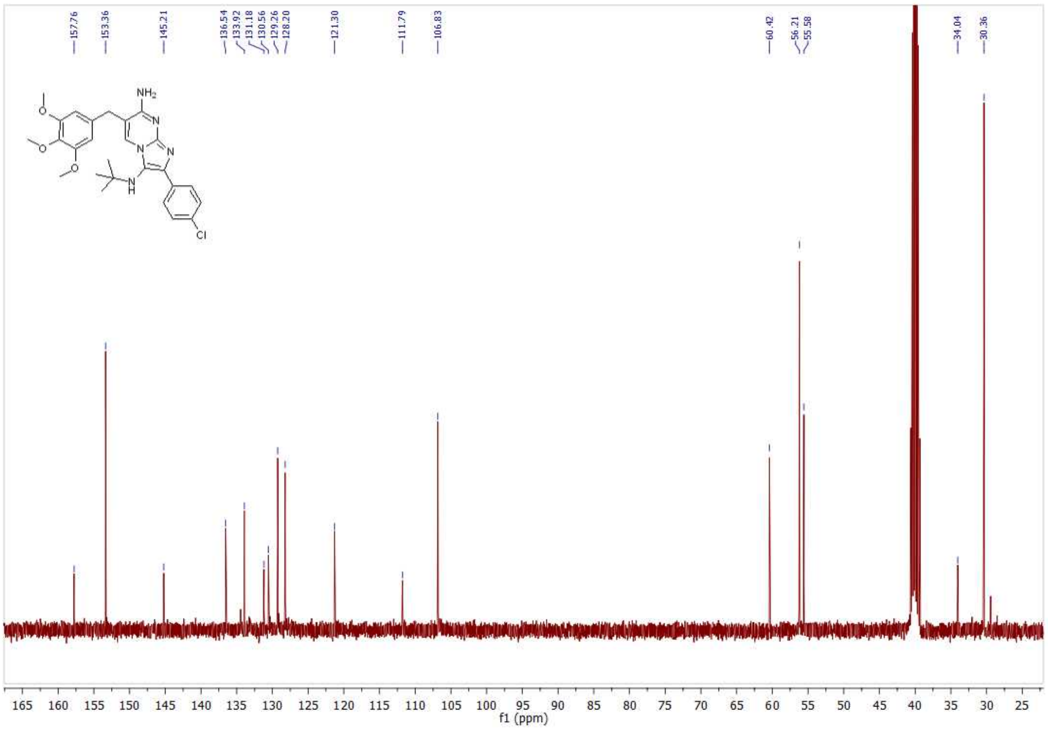

HMBC:

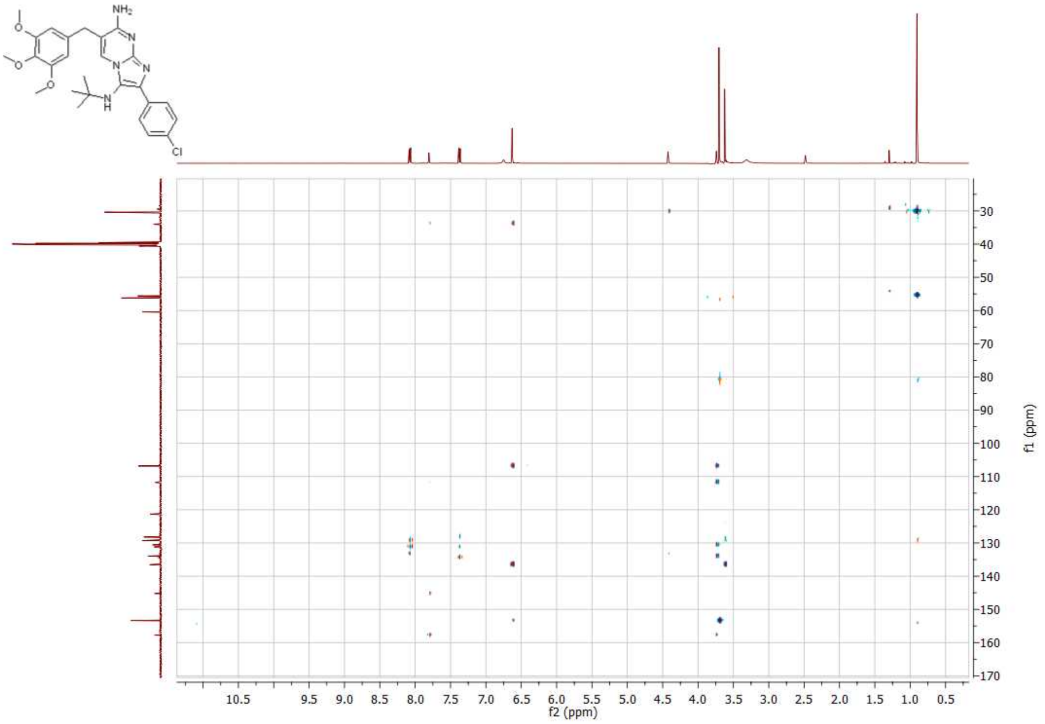

HSQC:

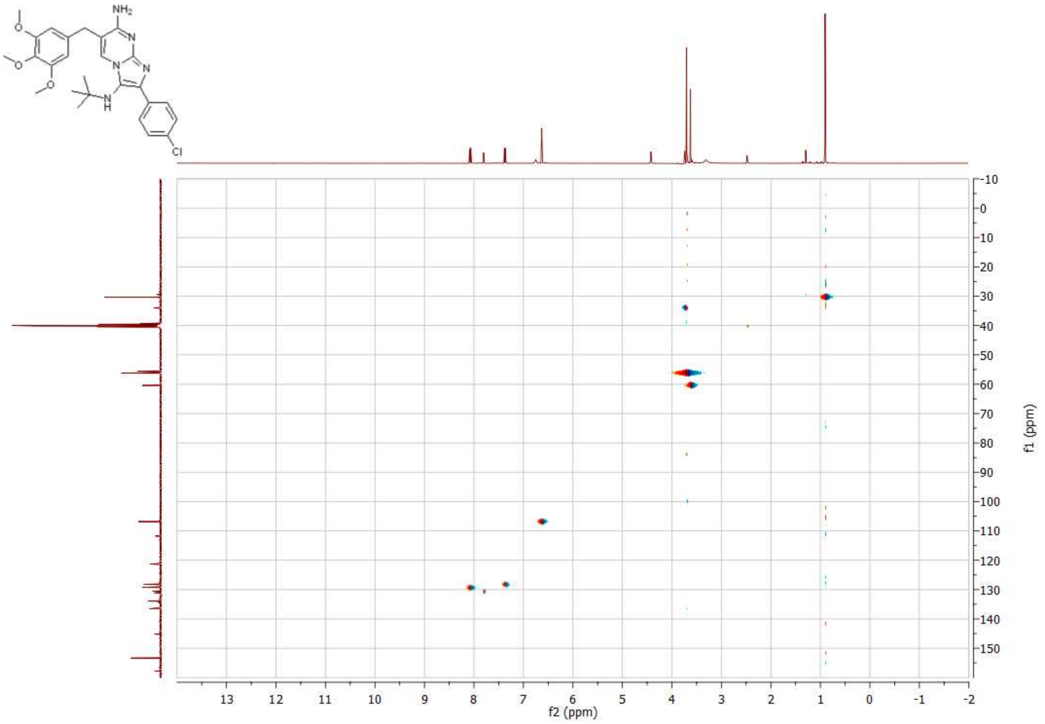

NOESY: Positive control is marked in blue, while diagnostic interactions are marked in red.

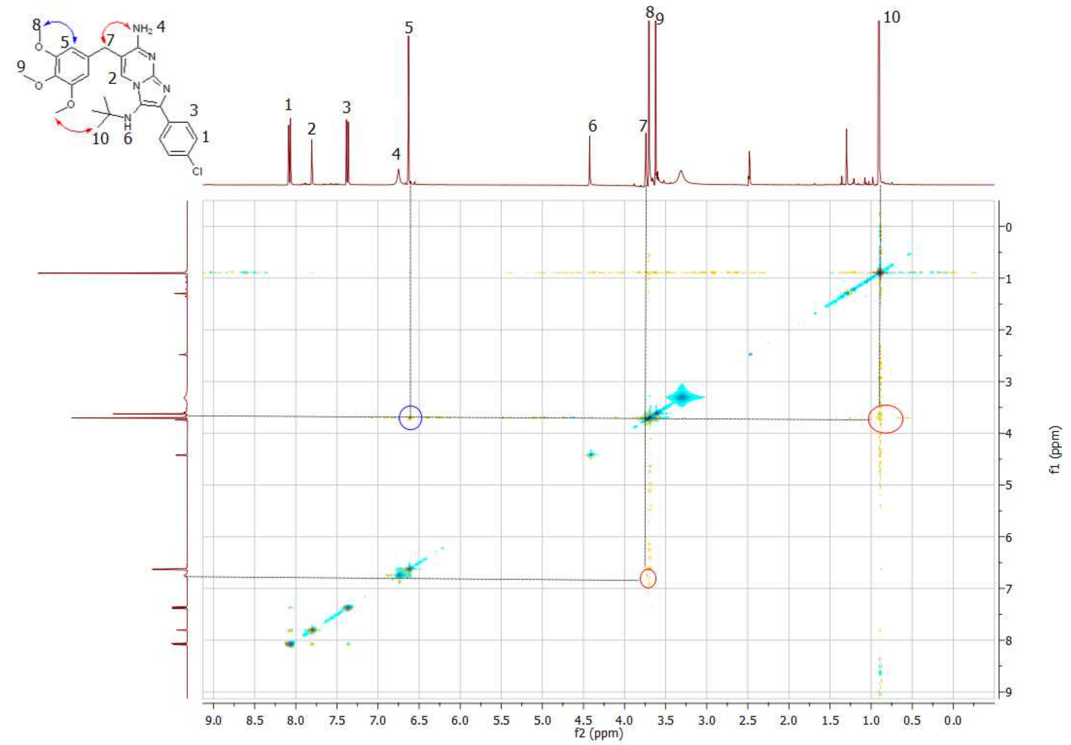

:

2-isopropyl-*N*[3]-(4-methoxyphenyl)-6-(3,4,5-trimethoxybenzyl)imidazo[1,2-*a*]pyrimidine-3,7-diamine (4b)

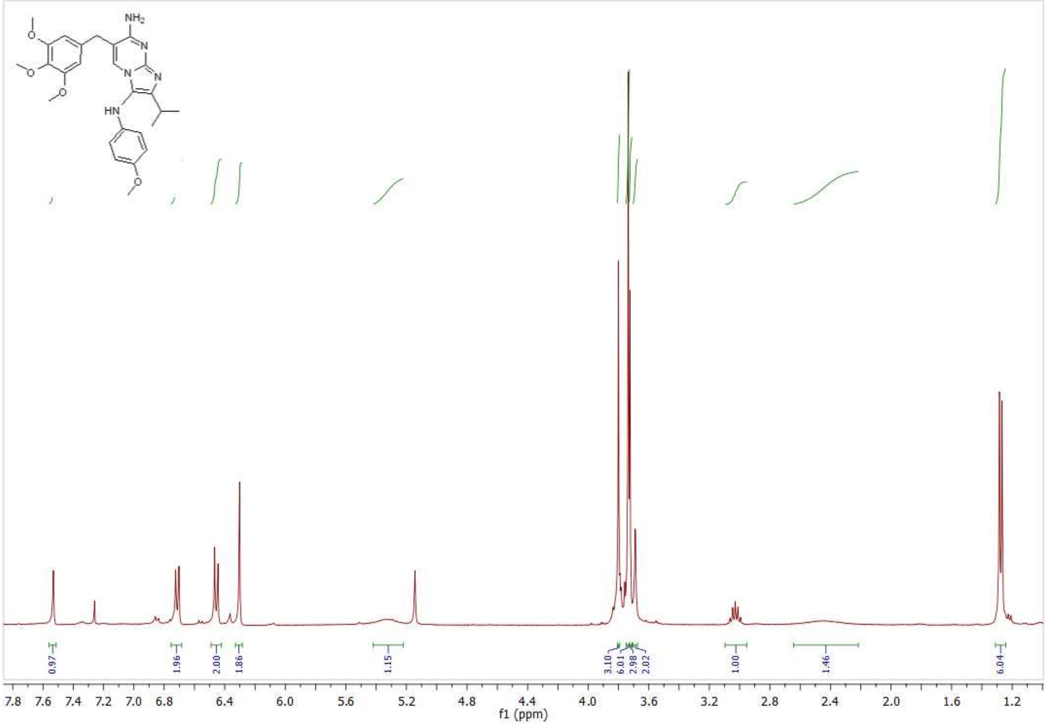

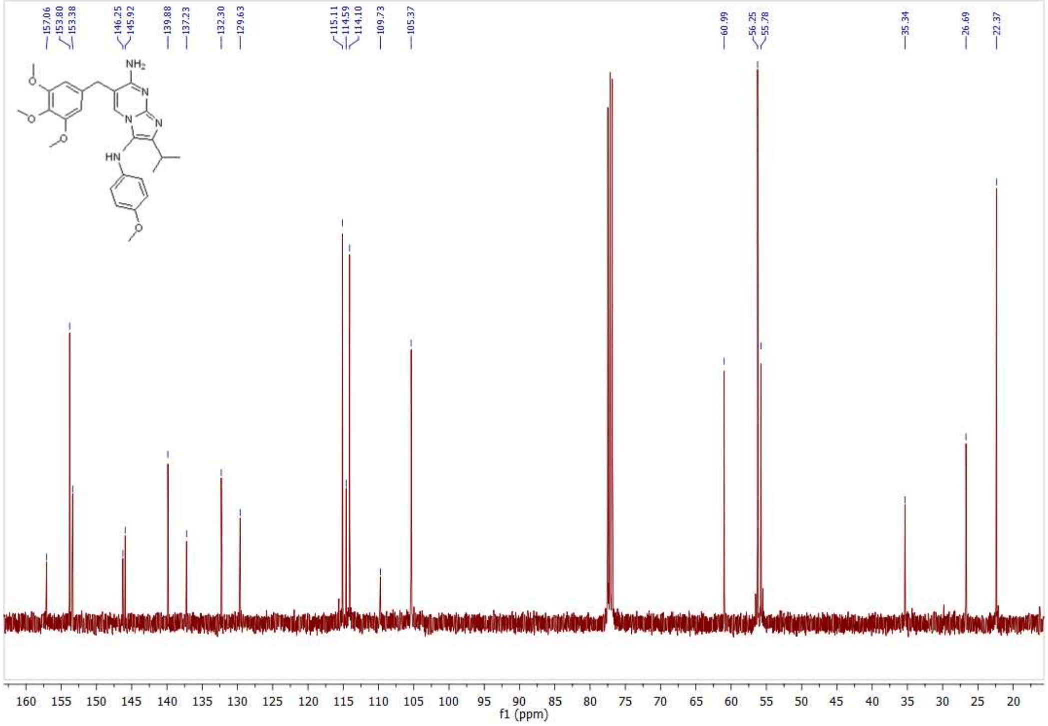

N^3-(tert-butyl)-2-isopropyl-6-(3,4,5-trimethoxybenzyl)imidazo[1,2-*a*]pyrimidine-3,7-diamine (4c)

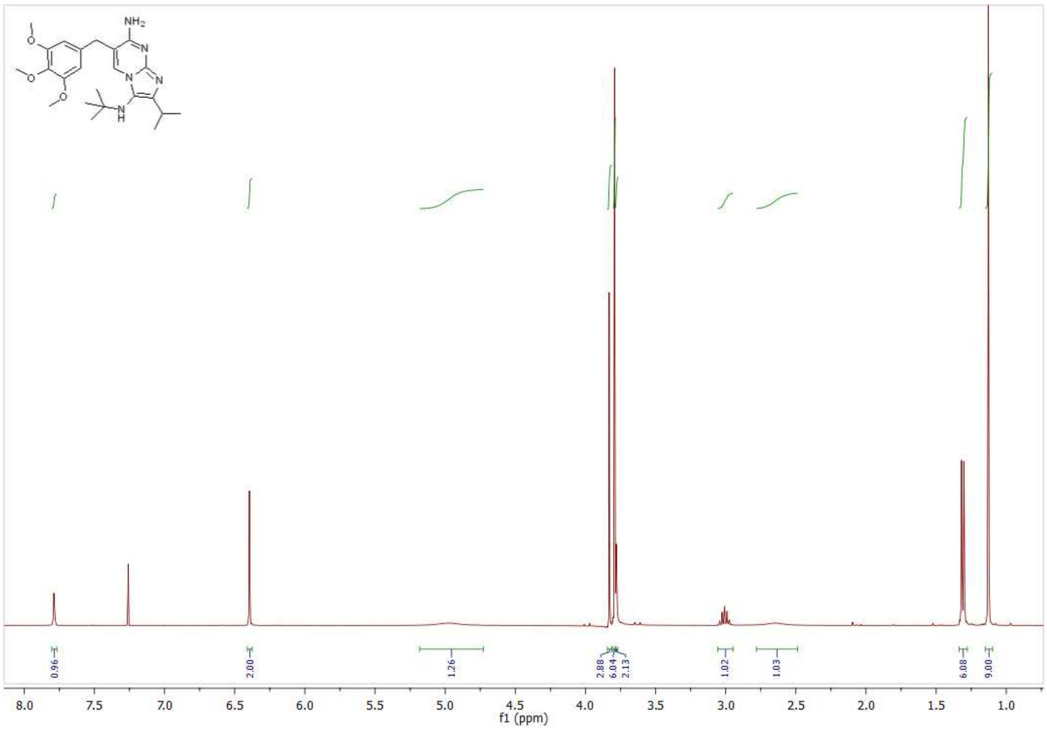

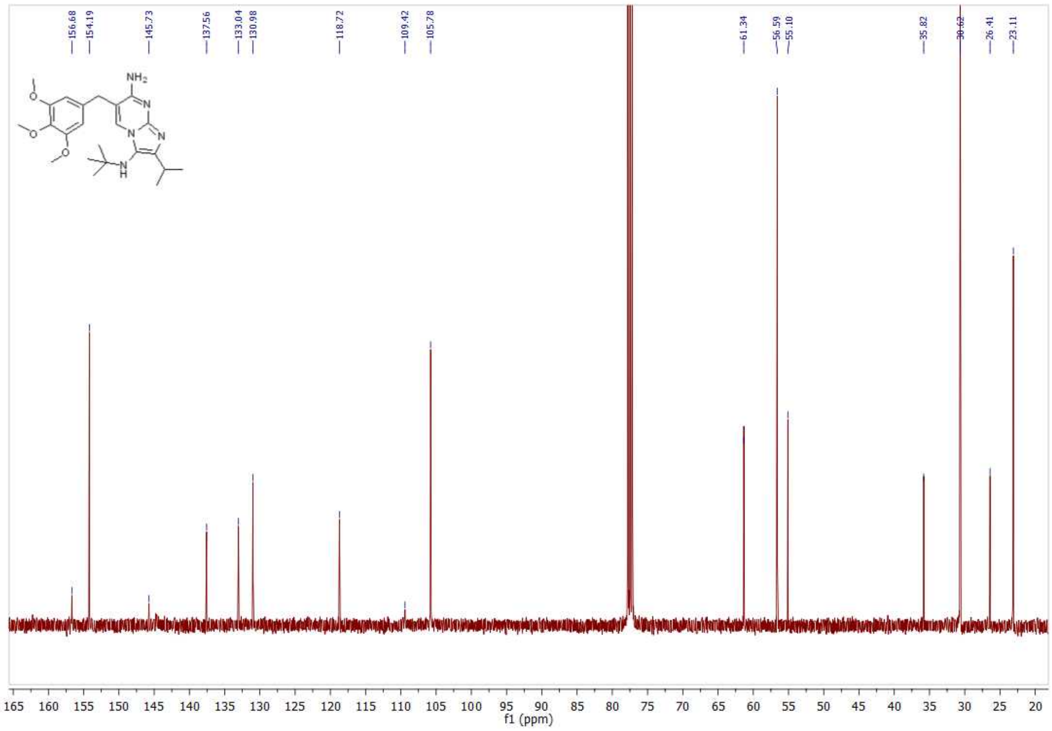

***N*[3]-cyclohexyl-2-(pyridin-2-yl)-6-(3,4,5-trimethoxybezyl)imidazo[1,2-*a*]pyrimidine-3,7-diamine (4d)**

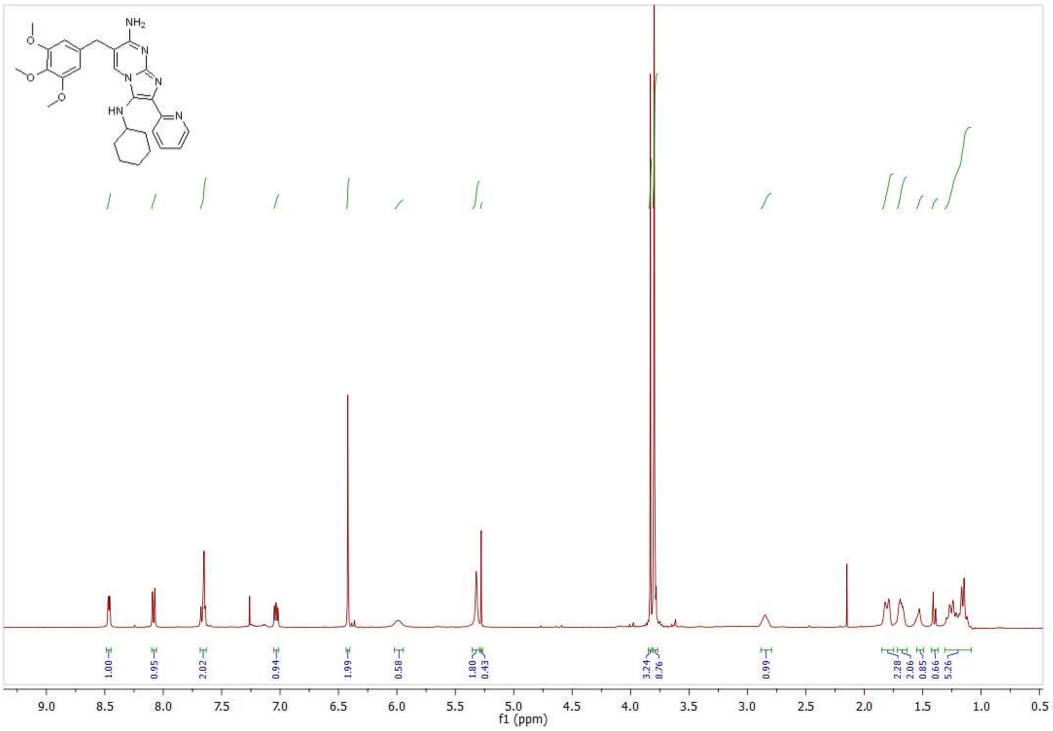

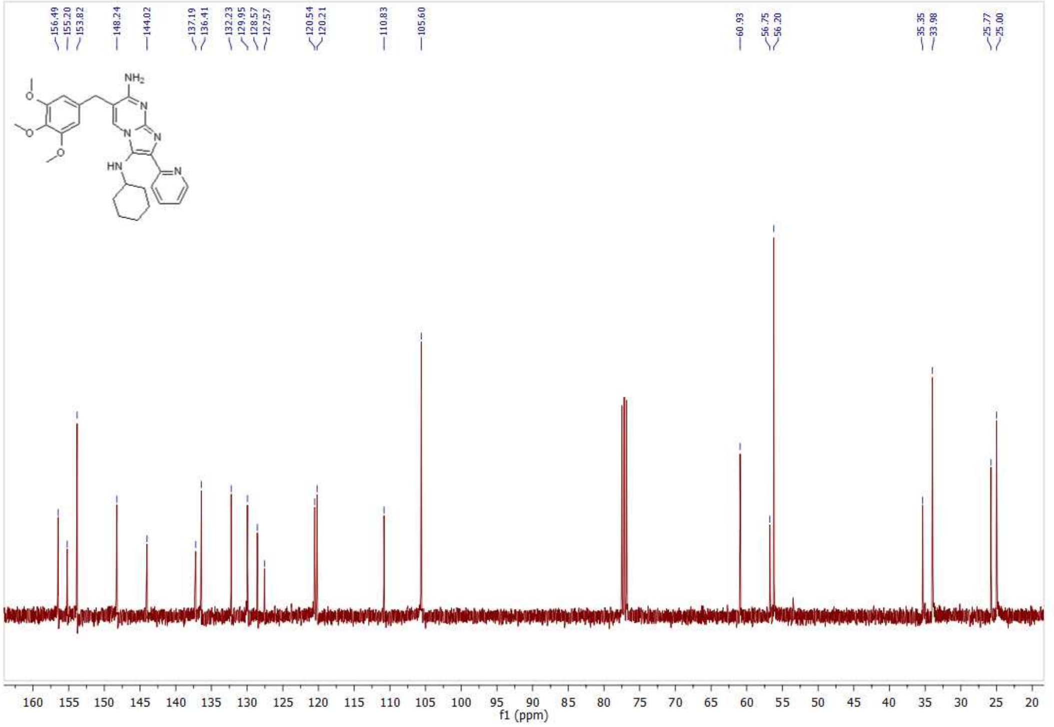

Ethyl(7-amino-2-(thiophen-2-yl)-6-(3,4,5-trimethoxybezyl)imidazo[1,2-*a*]pyrimidin-3-yl)glycinate (4e)

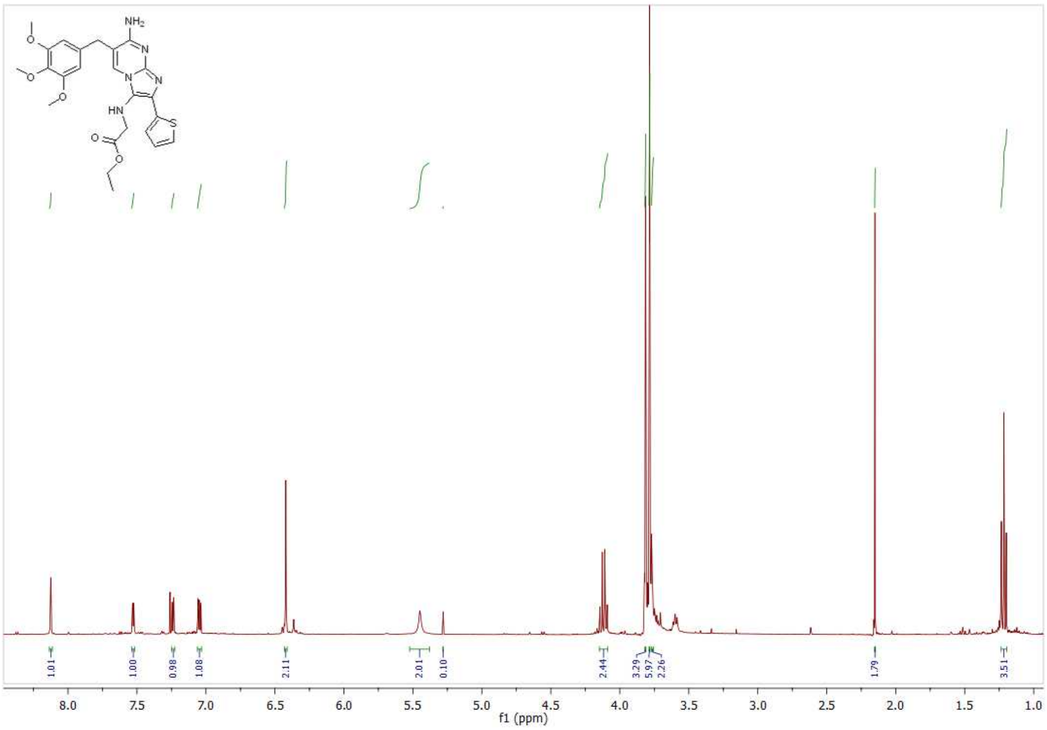

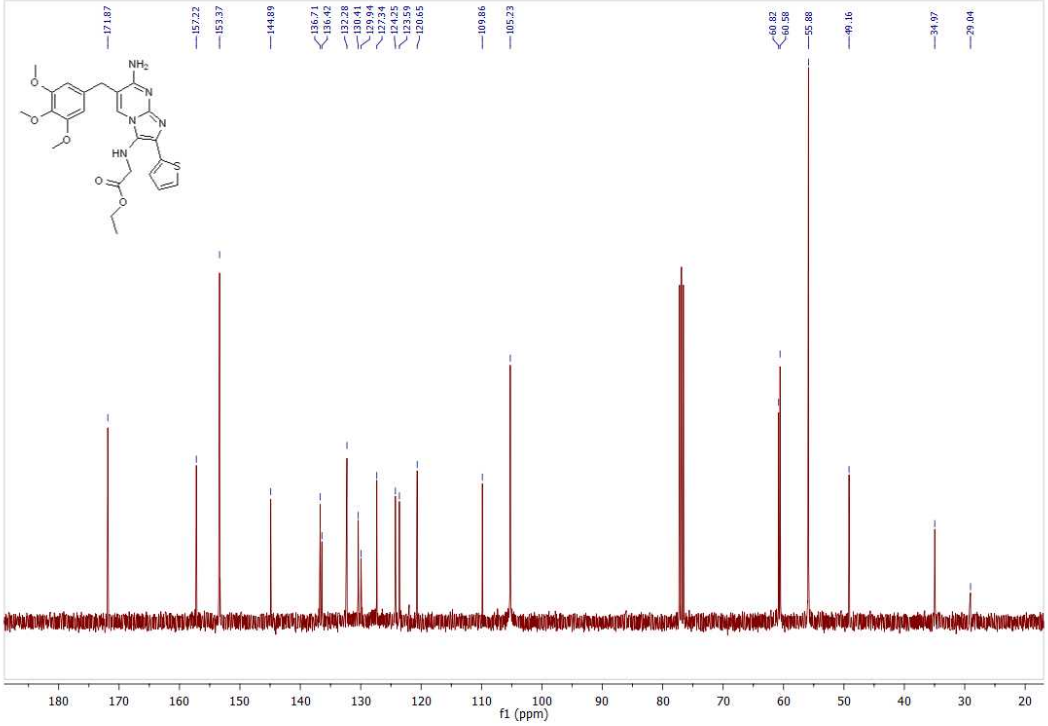

N^3-(*tert*-butyl)-2-methyl-6-(3,4,5-trimethoxybenzyl)imidazo[1,2-*a*]pyrimidine-3-7-diamine (4f)

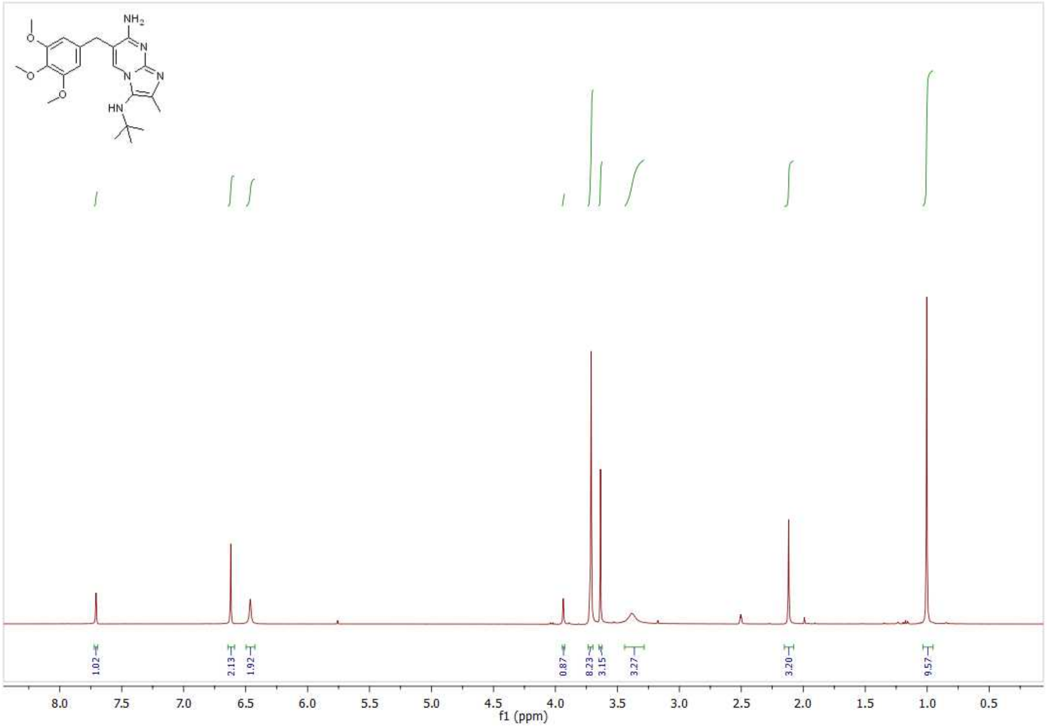

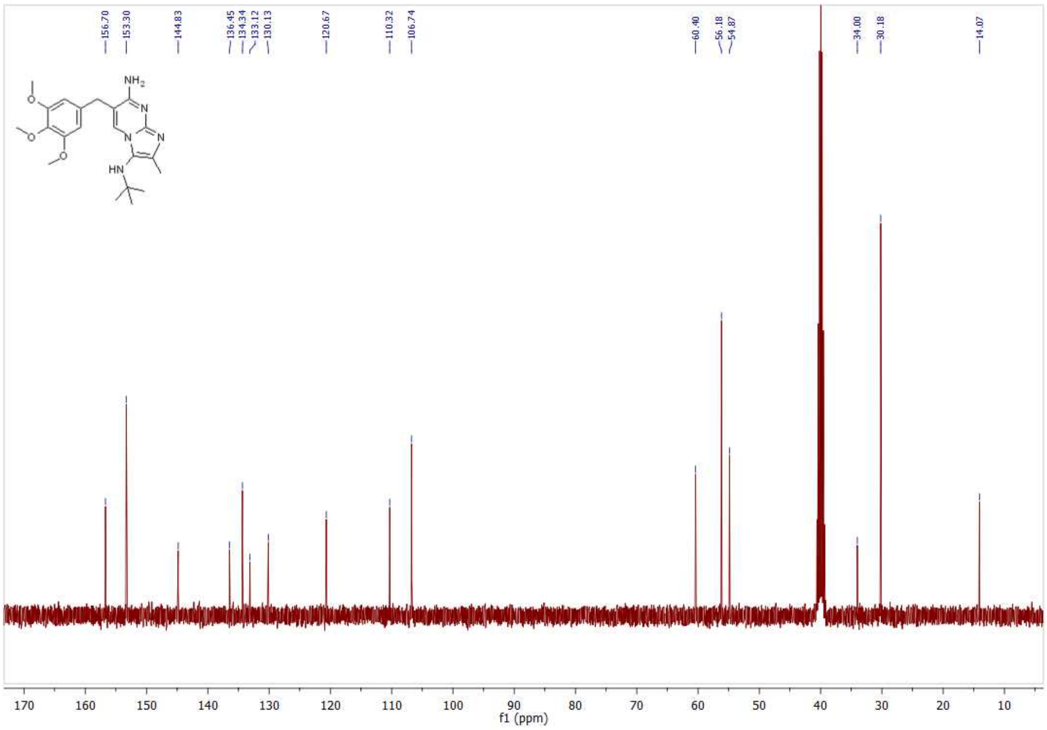

N^3-(*tert*-butyl)-2-(pyridin-2-yl)-6-(3,4,5-trimethoxybenzyl)imidazo[1,2-*a*]pyrimidine-3,7-diamine (4g)

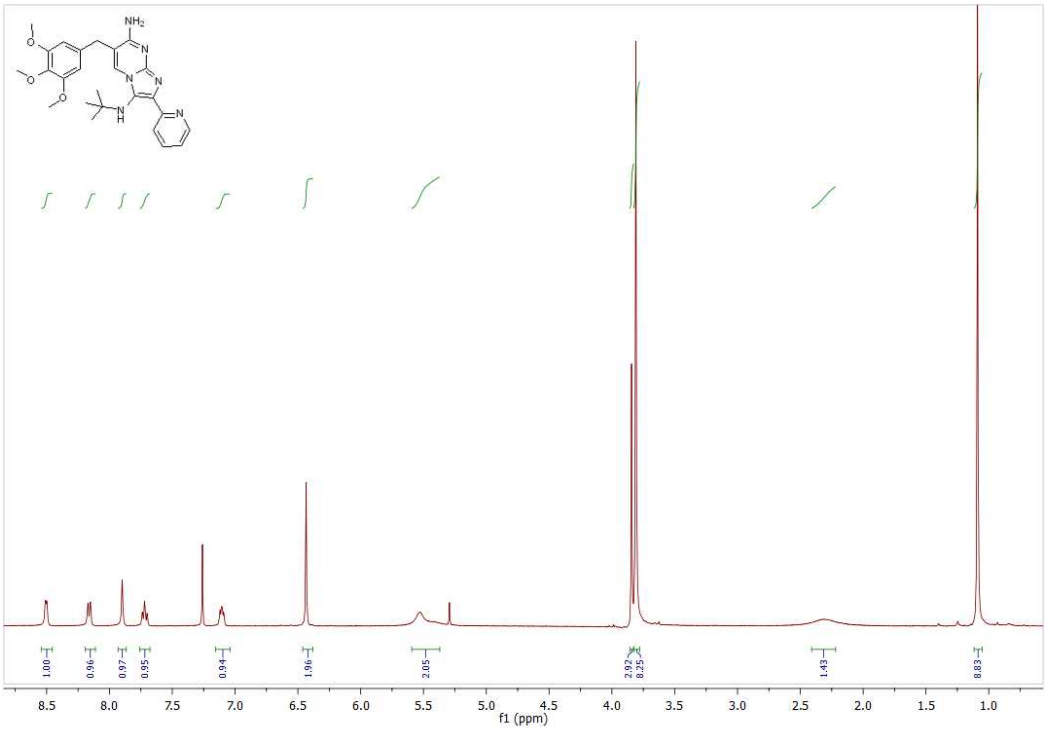

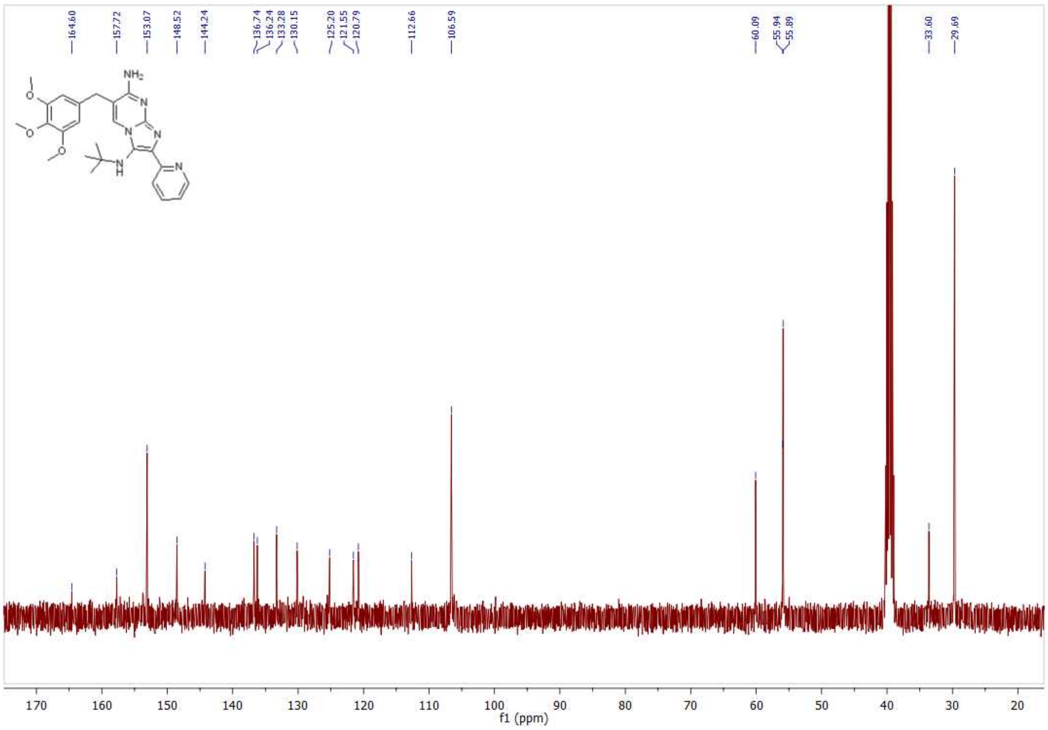

***N*[3]-(*tert*-butyl)-2-(thiophen-2-yl)-6-(3,4,5-trimethoxybenzyl)imidazo[1,2-*a*]pyrimidine-3,7-diamine (4h)**

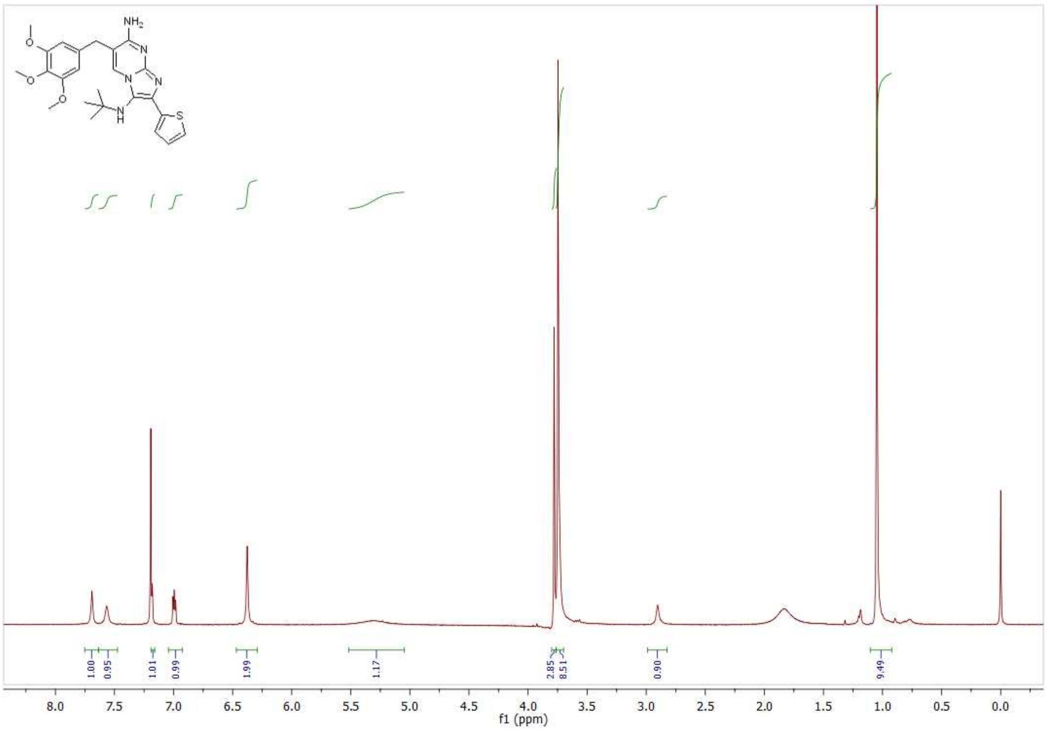

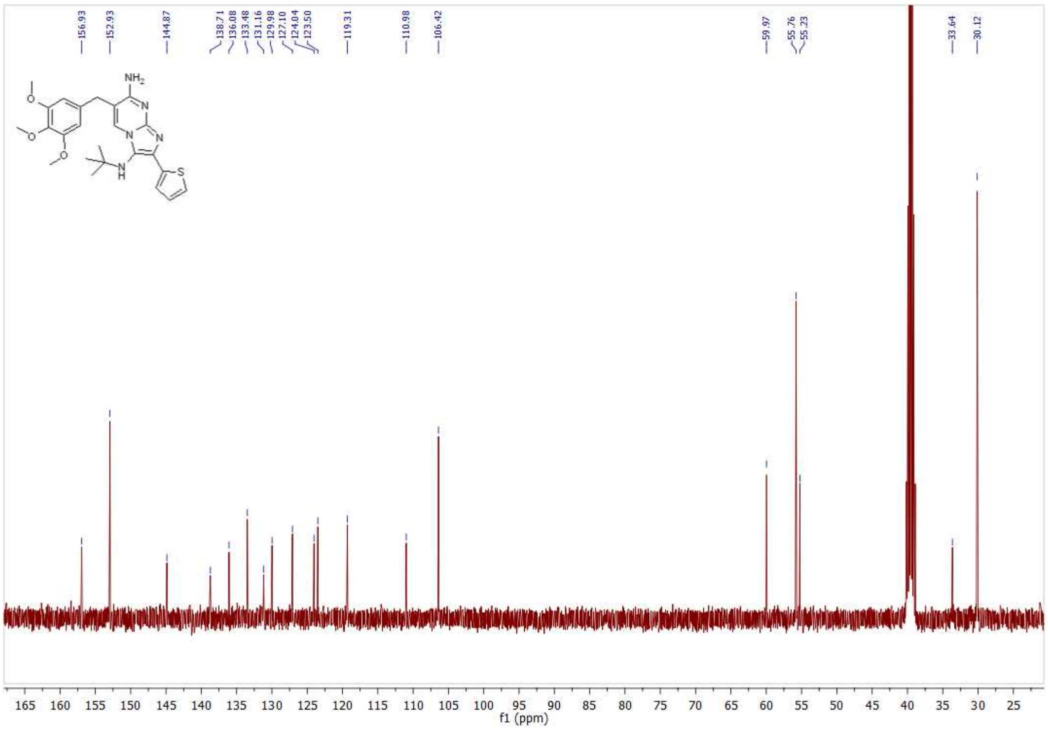

N^3-cyclohexyl-2-(pyridin-3-yl)-6-(3,4,5-trimethoxybenzyl)imidazo[1,2-*a*]pyrimidine-3,7-diamine (4i)

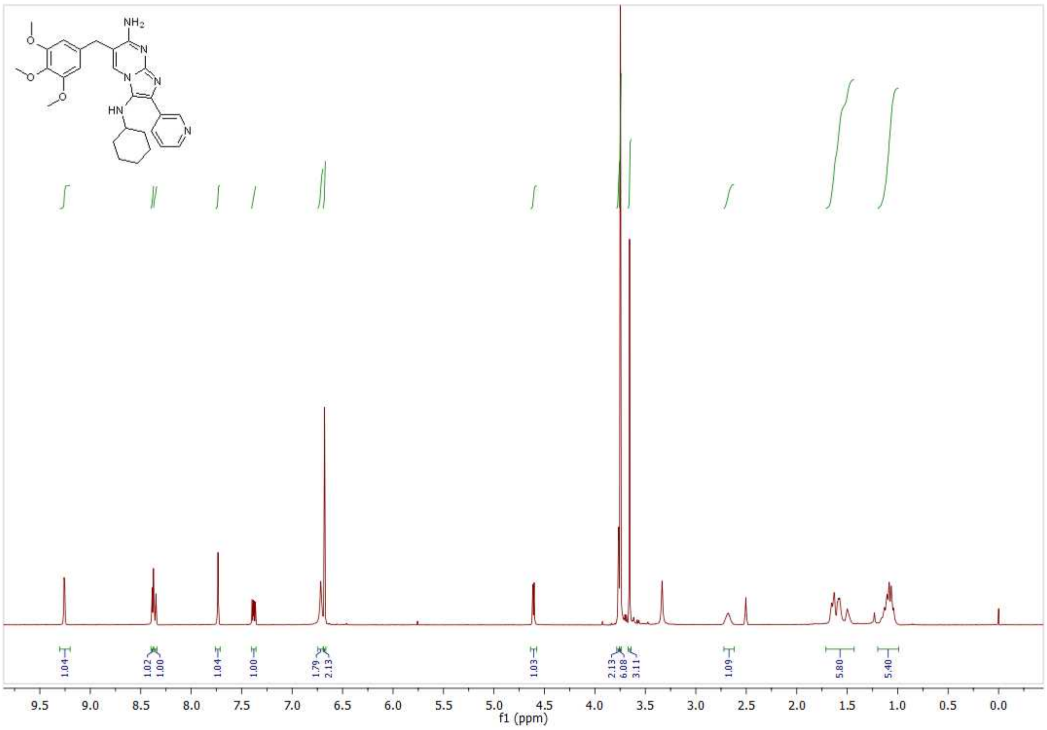

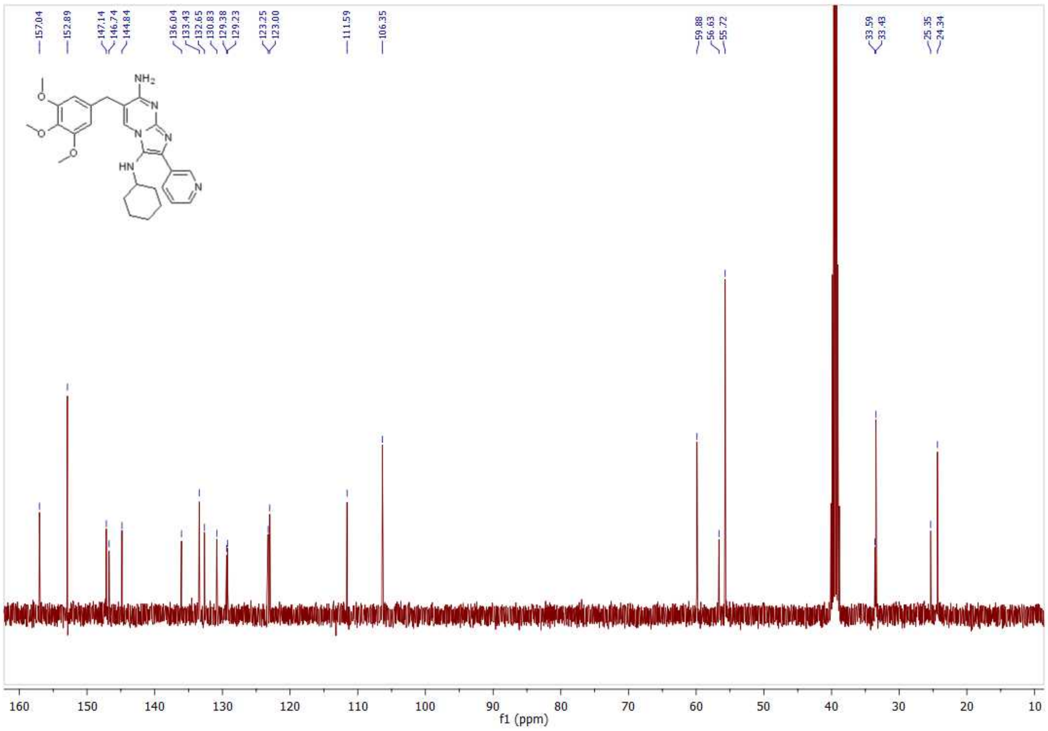

***N*[3]-cyclohexyl-2-(pyridine-4-yl)-6-(3,4,5-trimethoxybenzyl)imidazo[1,2-*a*]pyrimidine-3,7-diamine (4j)**

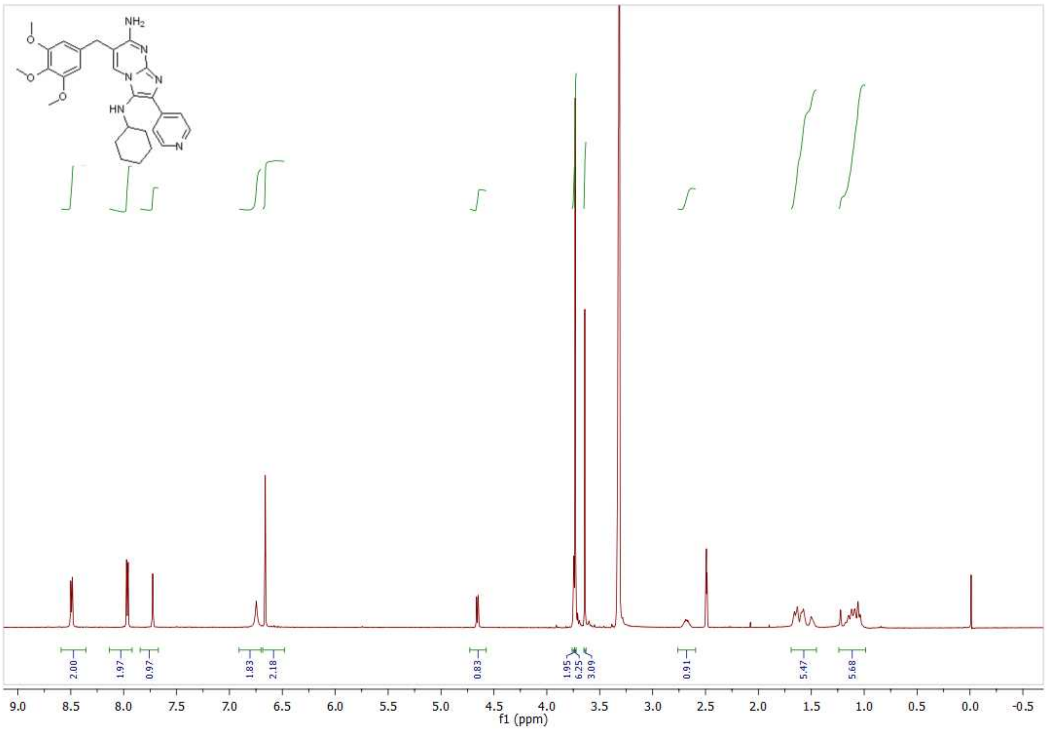

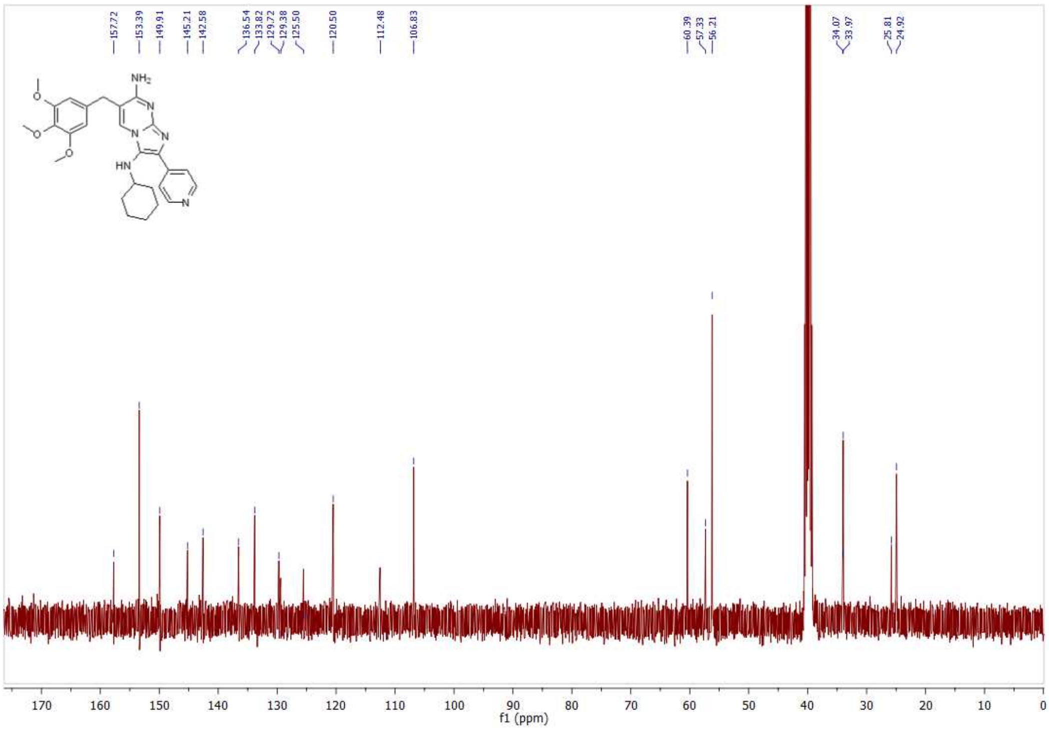

N^3,N^9-di-tert-butyl-2,8-bis(4-chlorophenyl)-6-(3,4,5-trimethoxybenzyl)diimidazo[1,2-*a*:1',2'-*c*]pyrimidine-3,9-diamine (5a)

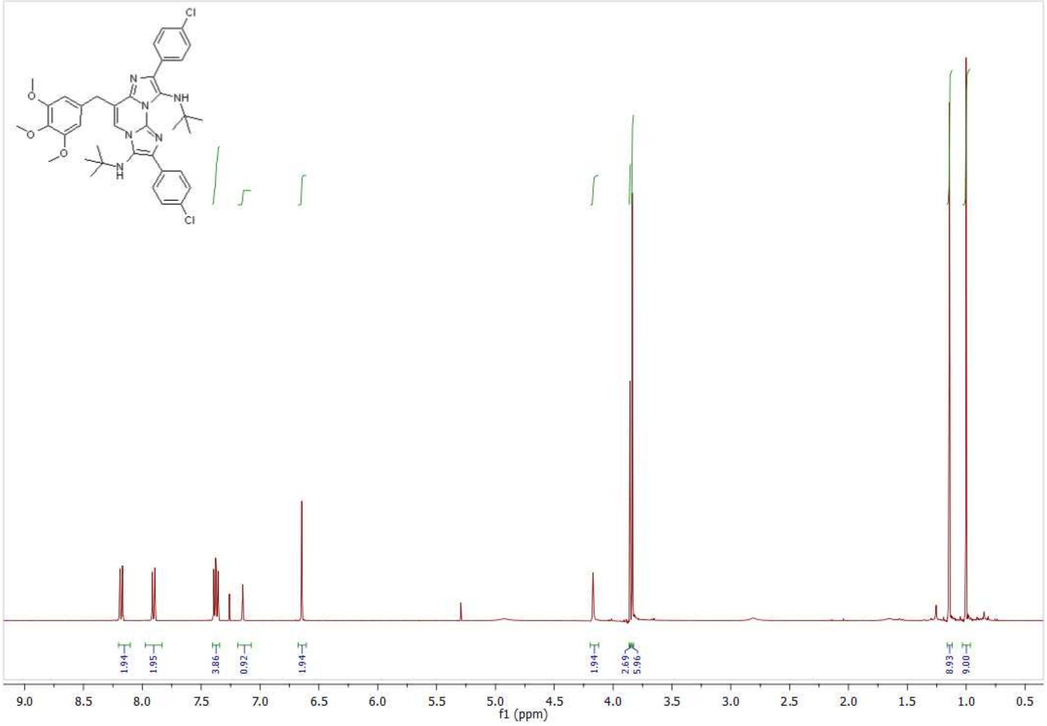

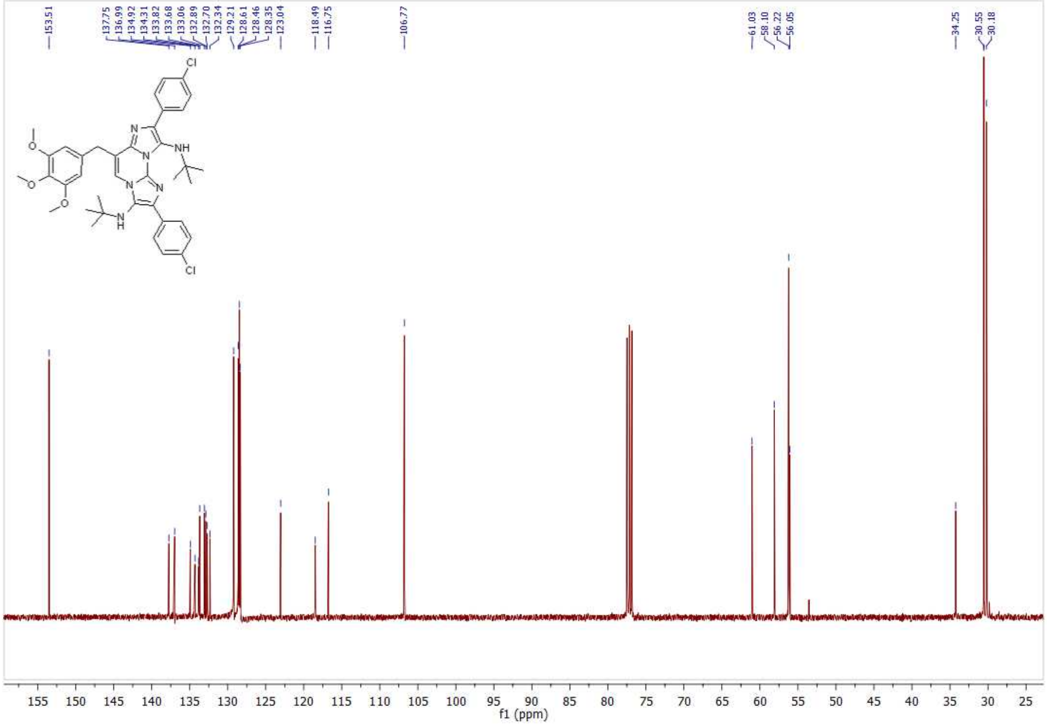

***N*^{3},*N*^{9}-di-tert-butyl-2,8-diisopropyl-6-(3,4,5-trimethoxybenzyl)diimidazo[1,2-*a*:1',2'-*c*]pyrimidine-3,9-diamine (5b)**

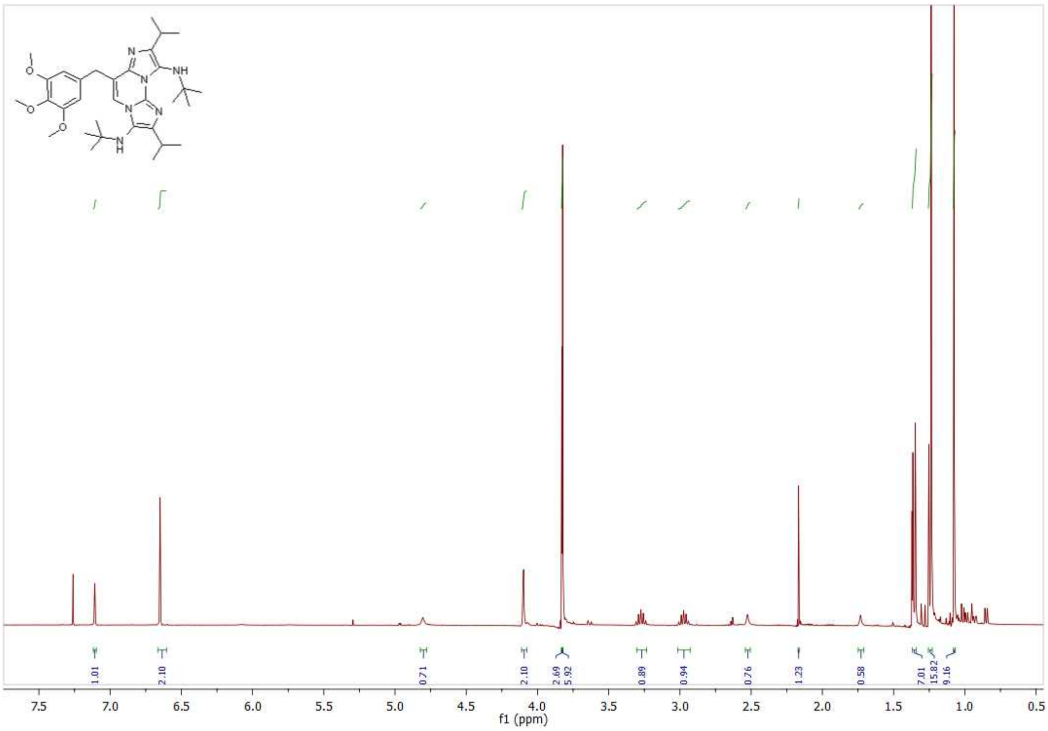

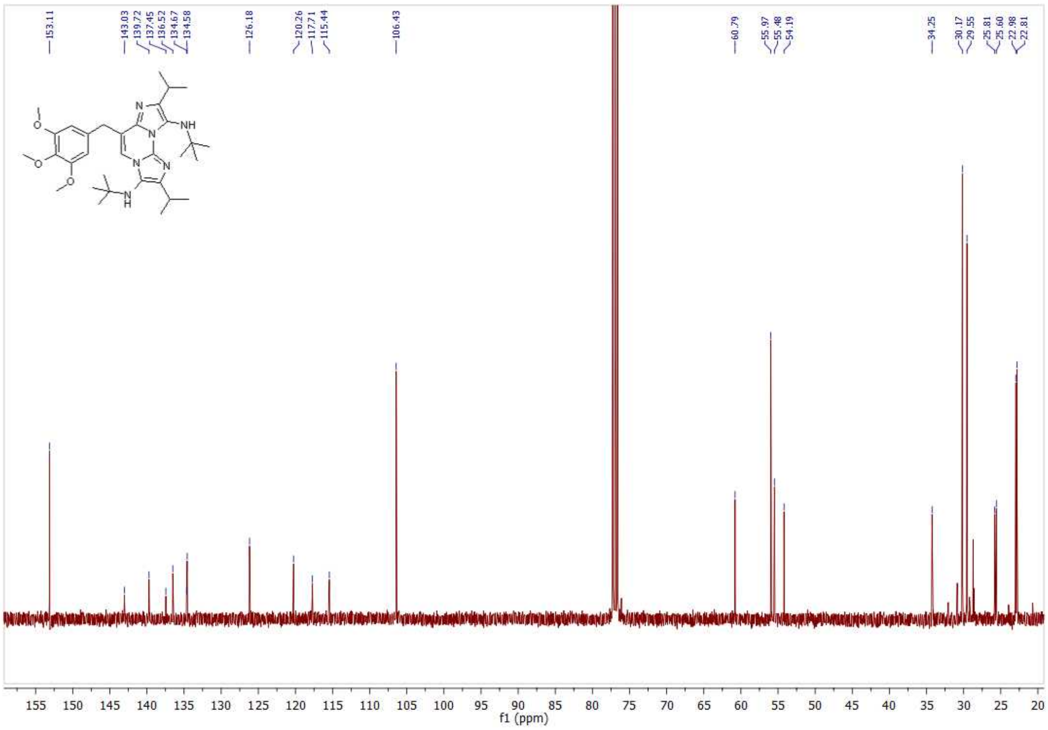

2-(4-chlorophenyl)-6-(3,4,5-trimethoxybenzyl)imidazo[1,2-*a*]pyrimidine-3,7-diamine (6a)

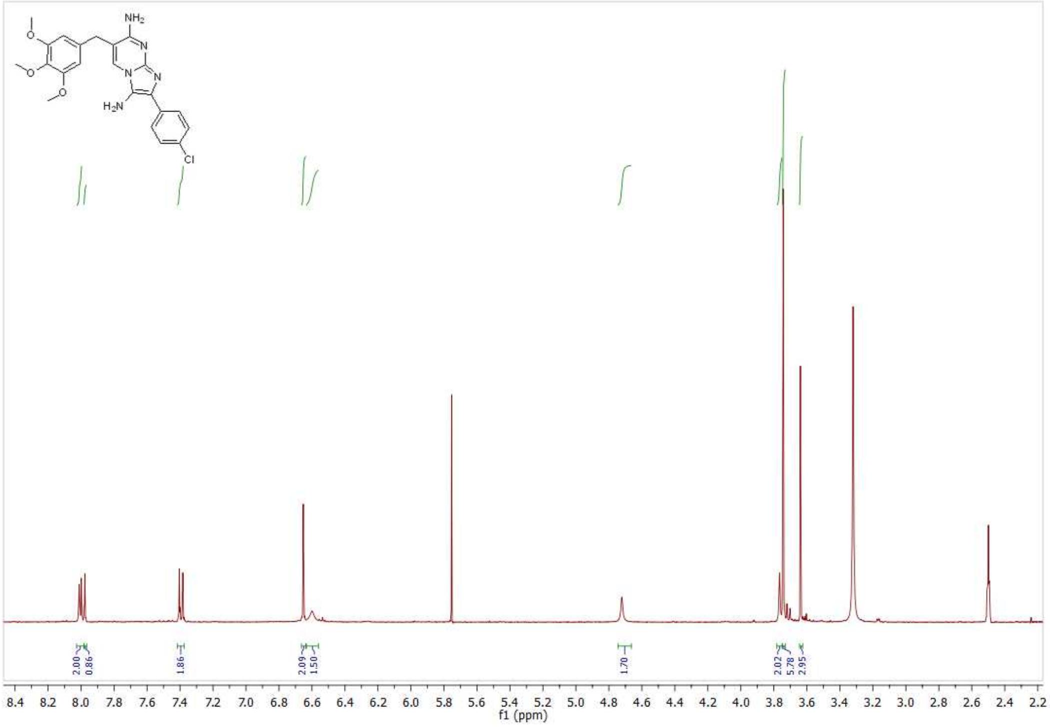

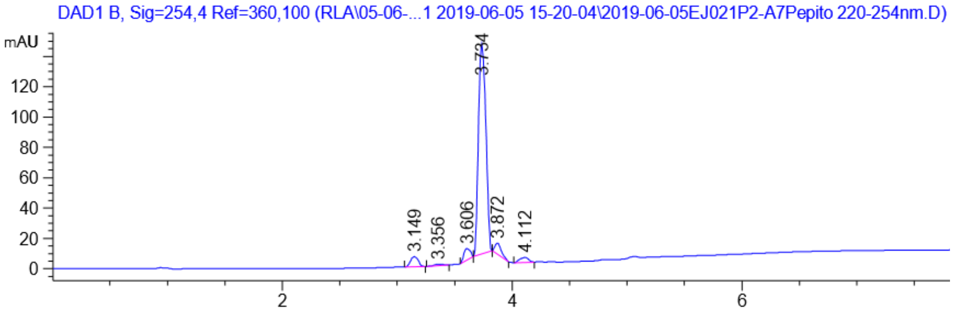

2-(pyridin-2-yl)-6-(3,4,5-trimethoxybenzyl)imidazo[1,2-*a*]pyrimidine-3,7-diamine (6b)

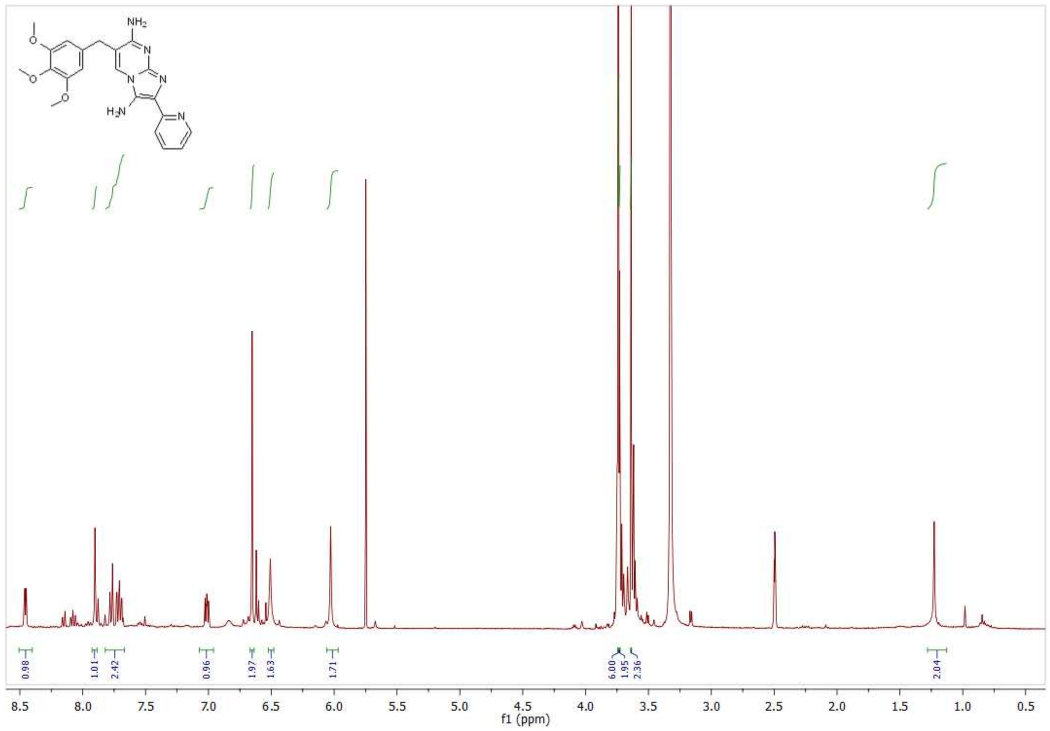

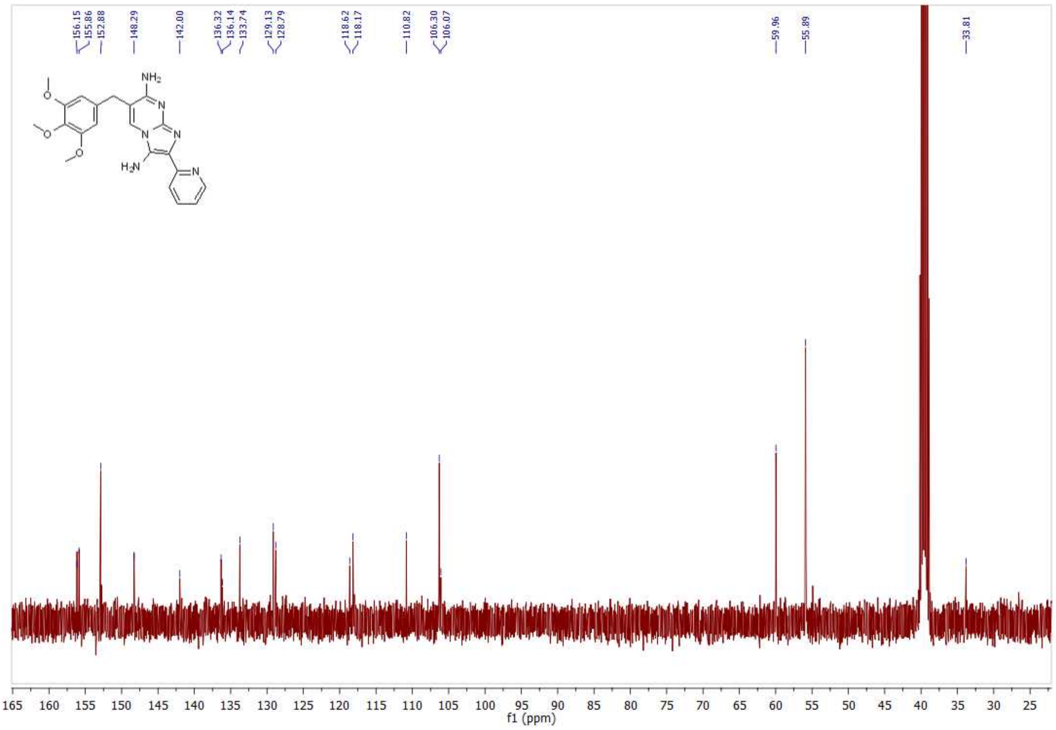

2,8-bis(4-chlorophenyl)-6-(3,4,5-trimethoxybenzyl)diimidazo[1,2-*a*:1',2'-*c*]pyrimidine-3,9-diamine (7a)

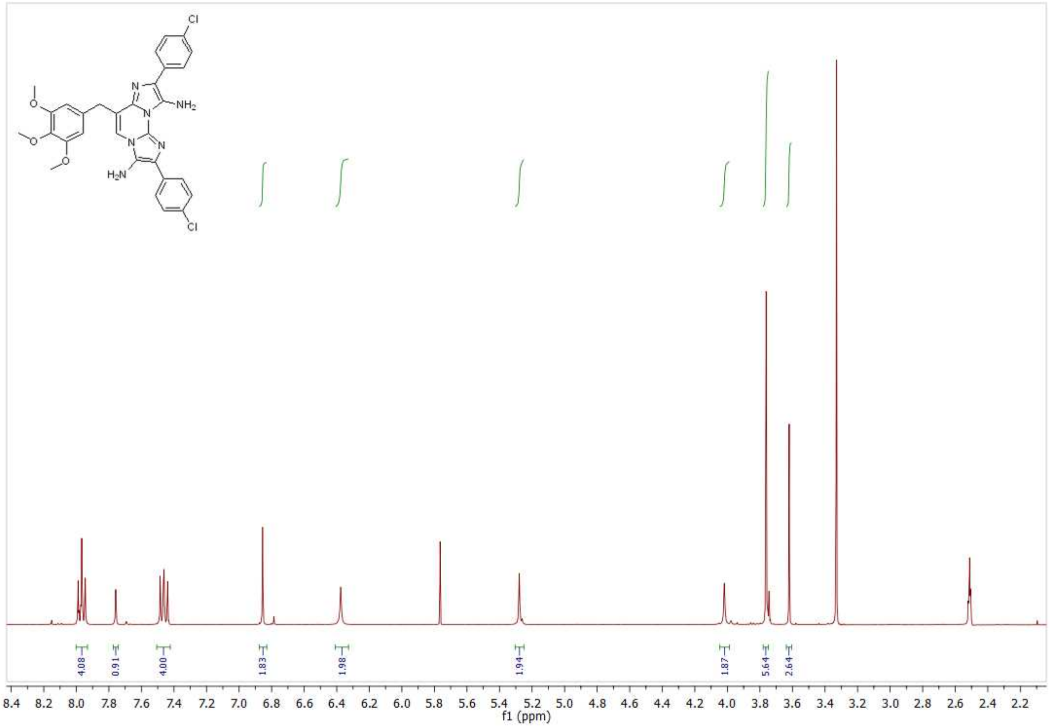

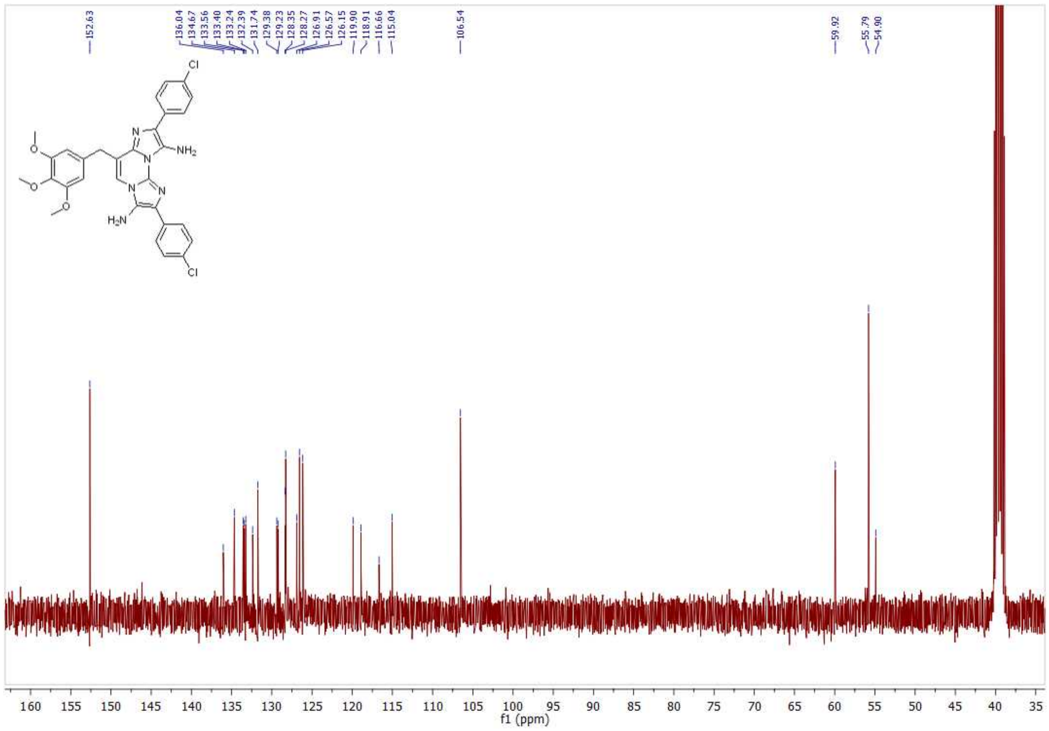

Microbiology

Material and Methods

Bacterial strains, bacteriological media and antimicrobials

Three different control strains were selected; *Escherichia coli* ATCC 25922, *Staphylococcus aureus* ATCC 29213 and *Pseudomonas aeruginosa* PAO1. Four clinical isolates of methicillin-resistant *S. aureus* from nasal and wound smear were used (*S. aureus* 8125304770, *S. aureus* 8139265926, *S. aureus* 8125255044, *S. aureus* 8124825998).

Tryptic soy broth (TSB, Scharlau Microbiology, Sentmenat, Spain) was used to determine the minimum inhibitory concentrations (MICs) and in the growth curves. Sulfamethoxazole and Trimethoprim were purchased from Sigma-Aldrich (St. Louis, USA).

Susceptibility testing

Determination of the Minimum Inhibitory Concentrations (MIC)

MIC values were determined by the broth microdilution method and interpreted according to the European Committee on Antimicrobial Susceptibility Testing (EUCAST) guidelines.[1] Briefly, the strains were grown overnight at 37 °C with orbital shaking at 200 rpm in Tryptone Soy Broth (TSB). After doing a refresh of 2 h in the same conditions, the bacterial cultures were adjusted to OD_{625nm} of 0.08–0.1 (10^8 CFUs/mL) in fresh TSB medium. 5 µL of each suspension was added to 96-well plates previously filled with TSB and serially diluted TMP analogues. Concentrations assayed were 32, 16, 8, 4, 2, 1, 0.5, 0.25, 0.125 and 0.062 µg/ml. The plates were incubated at 37 ºC for 24 h, after which the MIC was determined macroscopically, based on the visually turbidity of the wells by placing the plates on top of a viewing device in form of a stand with an enlarging mirror.

The antimicrobial activity of these 15 new Trimethoprim (TMP) analogues was evaluated against *Escherichia coli* ATCC 25922, *Staphylococcus aureus* ATCC 29213, *Pseudomonas aeruginosa* 01. Moreover, susceptibility was examined using the new compounds alone and in combination with Sulfamethoxazole in a 1:20 ratio in the three previous strains and also in *S. aureus* 8125304770, *S. aureus* 8139265926, *S. aureus* 8125255044 and *S. aureus* 8124825998. Serial dilutions of the antimicrobials starting from 32 µg/mL were tested.

Minimal inhibitory concentrations were determined in triplicate in at least three different experiments. Values resulted to be repetitive with undetectable differences by this method.

[1] EUCAST. European Committee on Antimicrobial Susceptibility Testing Breakpoint Tables for Interpretation of MICs and Zone Diameters European Committee on Antimicrobial Susceptibility Testing Breakpoint tables for Interpretation of MICs and Zone Diameters 0–99. Available online: http://www.eucast.org/fileadmin/src/media/PDFs/EUCAST_files/Breakpoint_tables/v_9.0_Breakpoint_Tables.pdf (accessed on 10 March 2019).

Effect of TMP-SMX and the GBBR analogues on bacterial growth curves

The combination of some of the most active analogues and Sulfamethoxazole (1:20) was assayed for their effect on the growth curve. A Gram-negative (*E. coli* ATCC 25922) and a Gram-positive (*S. aureus* ATCC 29213) were used to examine the effect of these new compounds in real time.

Volumes of 10 mL TSB liquid cultures with 1-5×10^8 CFU/mL in logarithmic-phase were adjusted. Antimicrobials were then added at sublethal concentrations (½ MIC and ¼ MIC). Trimethoprim was also evaluated as a control.

The incubation was performed in RTS-1C real-time cell growth loggers (Biosan) for 20h at 37 °C and 2000 rpm. Growth was measured as optical density (OD 850 nm) every 10 minutes.

Growth Curves

Effect of the analogues **4c**, **4d**, **4e**, **4f**, **4h**, **4j**, **6b** and **TMP** in combination with sulfamethoxazole (1:20) on the growth curve of *S. aureus*.

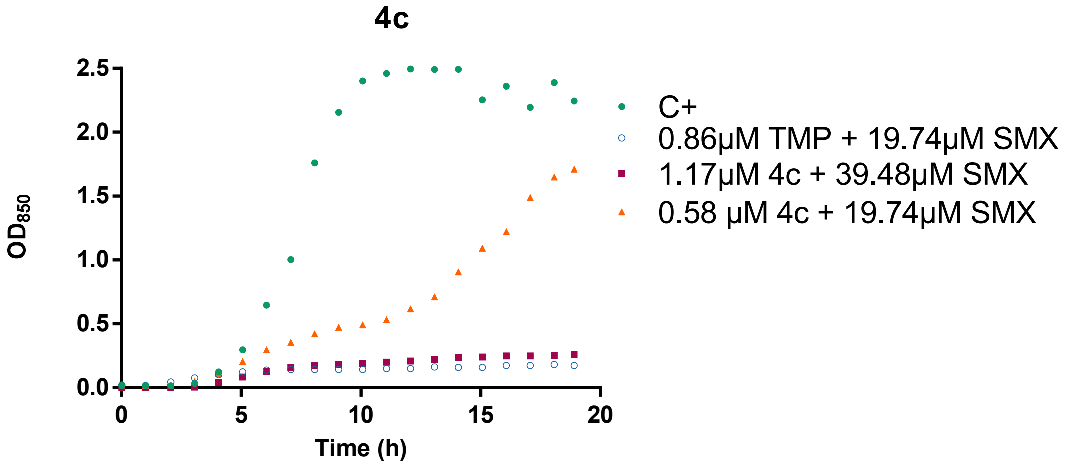

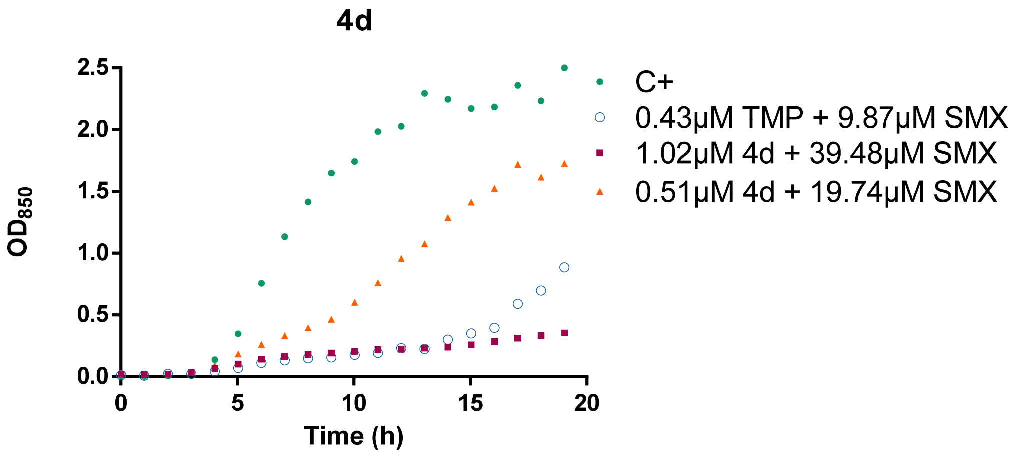

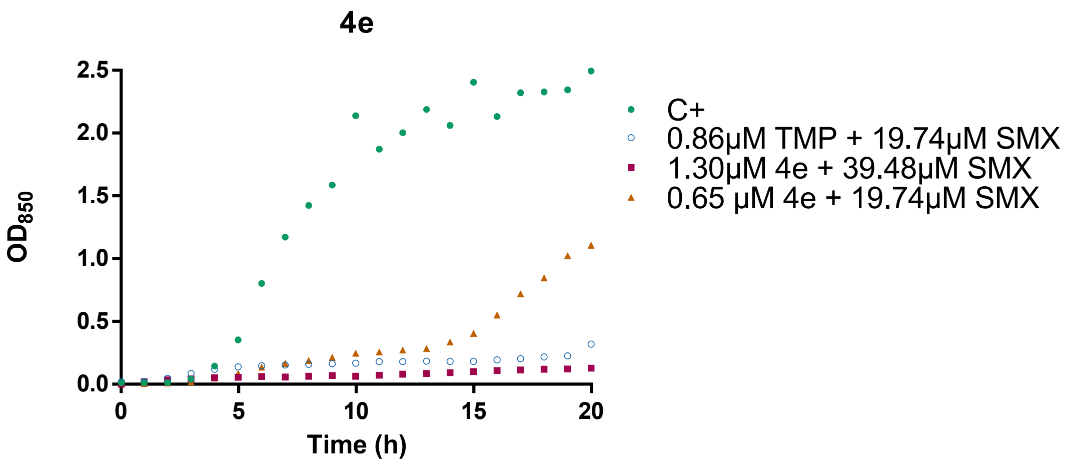
4e
OD850
2.5
2.0
1.5
1.0
0.5
0.0
0
5
10
15
20
Time (h)
C+
0.86μM TMP + 19.74μM SMX
1.30μM 4e + 39.48μM SMX
0.65 μM 4e + 19.74μM SMX

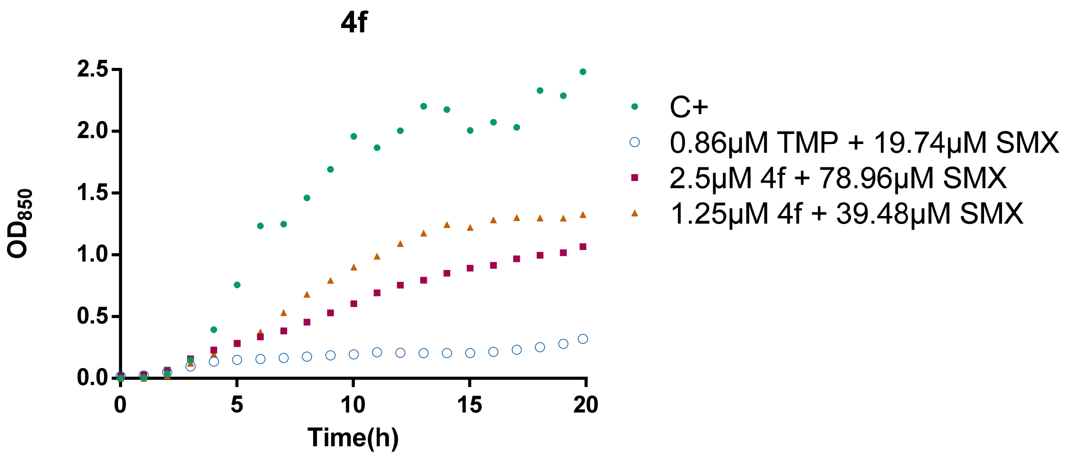
4f
OD850
2.5
2.0
1.5
1.0
0.5
0.0
0
5
10
15
20
Time(h)
C+
0.86μM TMP + 19.74μM SMX
2.5μM 4f + 78.96μM SMX
1.25μM 4f + 39.48μM SMX

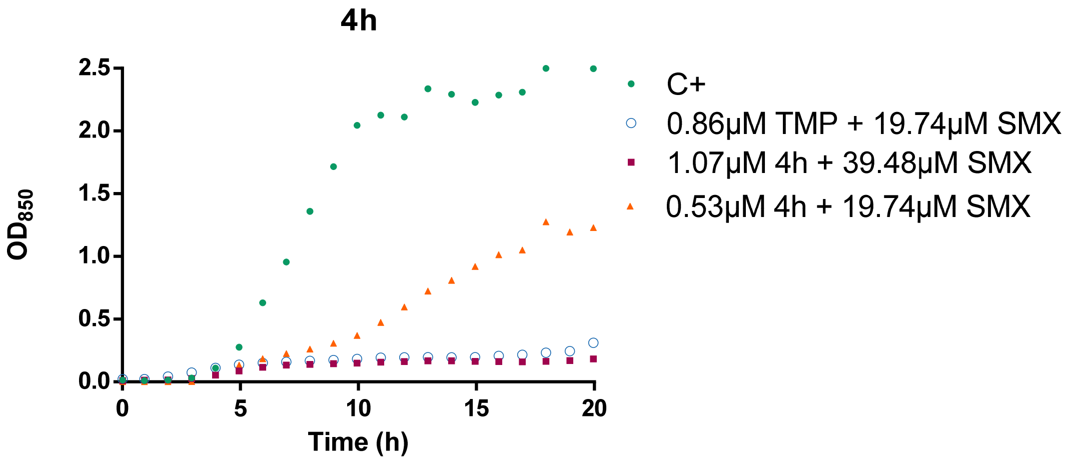
4h
OD850
2.5
2.0
1.5
1.0
0.5
0.0
0
5
10
15
20
Time (h)
C+
0.86μM TMP + 19.74μM SMX
1.07μM 4h + 39.48μM SMX
0.53μM 4h + 19.74μM SMX

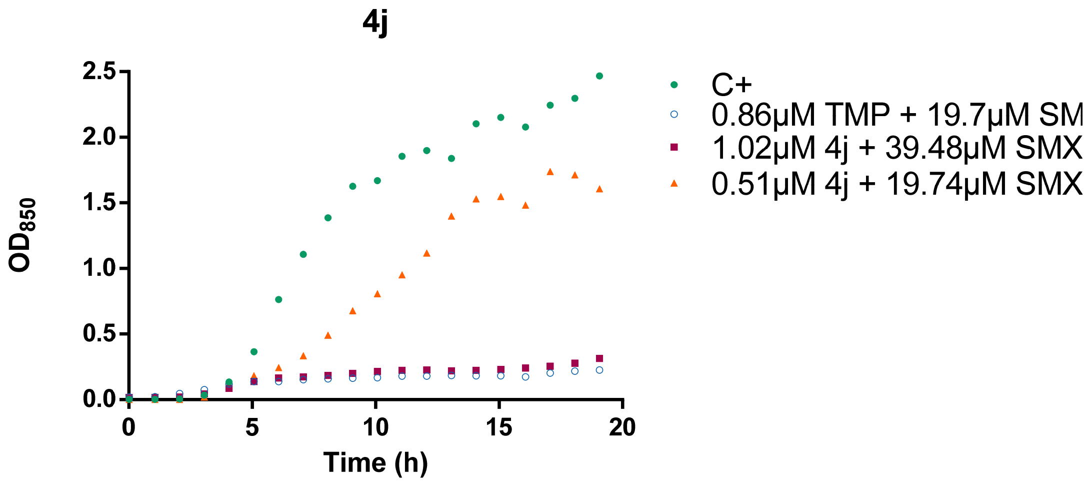
4j
OD850
2.5
2.0
1.5
1.0
0.5
0.0
0
5
10
15
20
Time (h)
C+
0.86µM TMP + 19.7µM SM
1.02µM 4j + 39.48µM SMX
0.51µM 4j + 19.74µM SMX

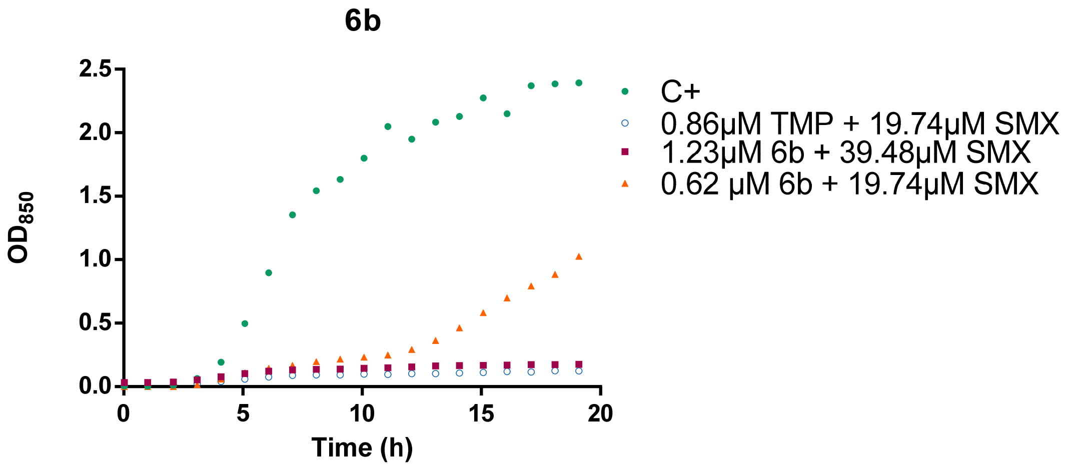
6b
OD850
2.5
2.0
1.5
1.0
0.5
0.0
0
5
10
15
20
Time (h)
C+
0.86µM TMP + 19.74µM SMX
1.23µM 6b + 39.48µM SMX
0.62 µM 6b + 19.74µM SMX

Effect of the analogues **4h** and **4i** and TMP in combination with sulfamethoxazole (1:20) on the growth curve of *E. coli*.

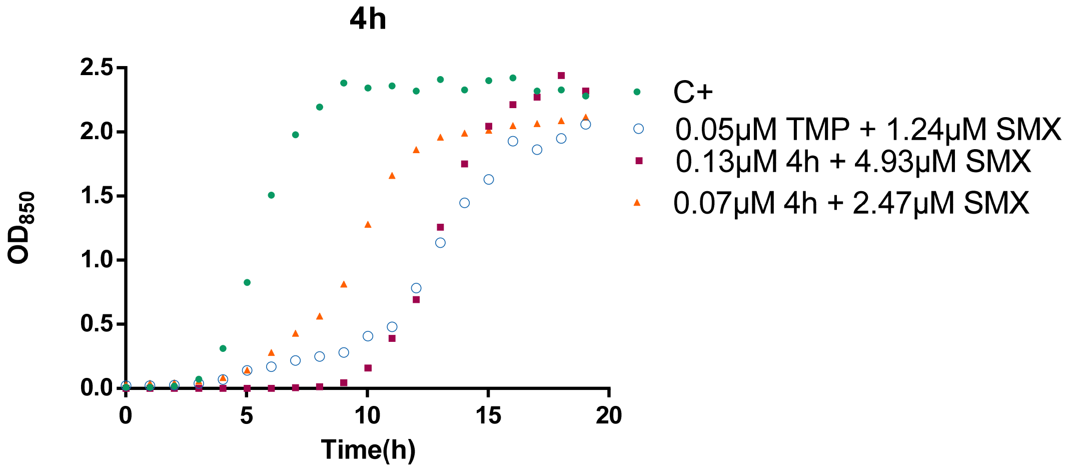

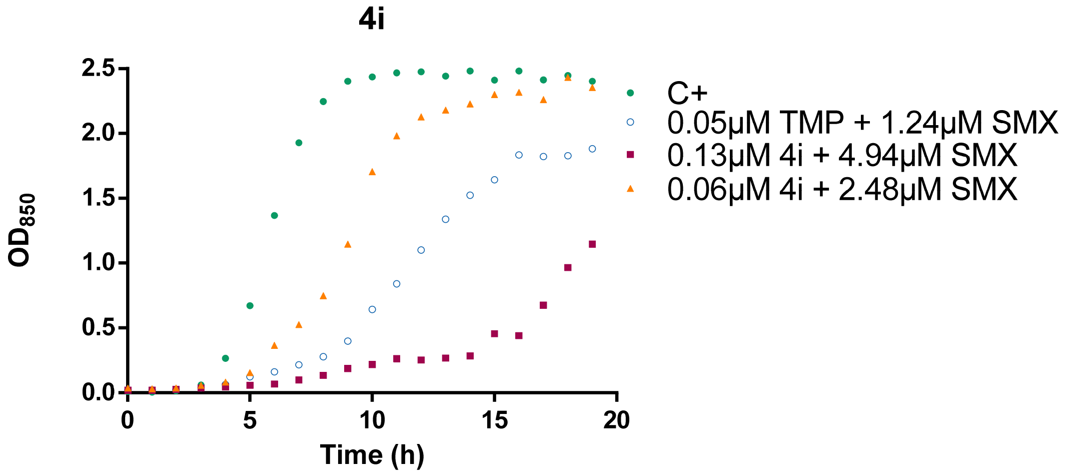

Supplement: Supplementary file 1 [file Data_Sheet_1.pdf]
